# Real‐world effectiveness and safety of oral azvudine versus nirmatrelvir‒ritonavir (Paxlovid) in hospitalized patients with COVID-19: a multicenter, retrospective, cohort study

**DOI:** 10.1038/s41392-025-02126-w

**Published:** 2025-01-17

**Authors:** Haiyu Wang, Guangying Cui, Ming Cheng, Tuerganaili Aji, Guotao Li, Xinjun Hu, Guangming Li, Shixi Zhang, Yanyang Zhang, Linqi Diao, Pan Li, Ling Wang, Yiqiang Yuan, Guowu Qian, Ruiqing Zhang, Xiaoli Jin, Juan Wang, Hong Luo, Donghua Zhang, Mingming Wang, Silin Li, Zhan Song, Mengzhao Yang, Guanyue Su, Ranran Sun, Junbiao Chang, Zujiang Yu, Zhigang Ren

**Affiliations:** 1 Department of Infectious Diseases, State Key Laboratory of Antiviral Drugs, Pingyuan Laboratory, the First Affiliated Hospital of Zhengzhou University, Zhengzhou, 450052 China; 2 Department of Medical Information, the First Affiliated Hospital of Zhengzhou University, Zhengzhou, 450052 China; 3 Department of Hepatobiliary and Echinococcosis Surgery, Digestive and Vascular Surgery Center, First Affiliated Hospital of Xinjiang Medical University, Urumqi, 830054 China; 4 Department of Infectious Diseases, Luoyang Central Hospital Affiliated of Zhengzhou University, Luoyang, 471000 China; 5 Department of Infectious Diseases, the First Affiliated Hospital, College of Clinical Medicine, Henan University of Science and Technology, Luoyang, 471003 China; 6 Department of Liver Disease, the Affiliated Infectious Disease Hospital of Zhengzhou University, Zhengzhou, 450052 China; 7 Department of Infectious Diseases, Shangqiu Municipal Hospital, Shangqiu, 476000 China; 8 Henan Center for Disease Control and Prevention, Zhengzhou, 450016 China; 9 Department of Cardiovascular Medicine, Henan Provincial Chest Hospital Affiliated of Zhengzhou University, Zhengzhou, 450008 China; 10 Department of Gastrointestinal Surgery, Nanyang Central Hospital, Nanyang, 473009 China; 11 Guangshan County People’s Hospital, Guangshan County, Xinyang, 465450 China; 12 Department of Infectious Diseases, Anyang City Fifth People’s Hospital, Anyang, 455000 China; 13 Department of Respiratory and Critical Care Medicine, Fengqiu County People’s Hospital, Xinxiang, 453300 China; 14 State Key Laboratory of Antiviral Drugs, Pingyuan Laboratory, Zhengzhou University, Zhengzhou, 450001 China

**Keywords:** Infectious diseases, Drug development

## Abstract

Azvudine and nirmatrelvir-ritonavir (Paxlovid) were widely used to treat patients with COVID-19 in China during the Omicron wave. However, the efficacy and safety of azvudine versus Paxlovid are poorly established. This study included 40,876 hospitalized patients with COVID-19 from eleven hospitals in Henan and Xinjiang Provinces, China. Clinical outcomes were compared between the two drugs via Kaplan–Meier analysis and Cox regression models. Additionally, in vitro and in vivo experiments were used to evaluate the antitumor effects and safety of both drugs. Single-cell RNA sequencing was performed to elucidate the tumor immune landscape after azvudine treatment. After propensity score matching, 2404 azvudine and 1202 Paxlovid recipients from Henan Province were included. Cox regression revealed that azvudine was related to an 18% lower risk of all-cause death than Paxlovid (95% CI: 0.676–0.987), was not obviously different in composite disease progression. The robustness of the findings was verified by the Xinjiang cohort and three sensitivity analyses. Fewer adverse events were observed in the azvudine group. Subgroup analysis revealed that azvudine provided greater benefits for patients with malignant tumors, significantly reducing both all-cause death (hazard ratio [HR]: 0.33, 95% CI: 0.20−0.54) and composite disease progression (HR: 0.54, 95% CI: 0.33−0.88). Furthermore, azvudine can suppress the growth of hepatocellular carcinoma (HCC) by regulating CD4^+^ T and CD8^+^ T cells in vivo. These findings suggest that azvudine therapy is not inferior to Paxlovid in hospitalized COVID-19 patients and has fewer adverse effects. Notably, azvudine may offer greater clinical benefit for patients with HCC.

## Introduction

Since the coronavirus disease 2019 (COVID-19) outbreak in December 2019, a highly infectious viral disease caused by severe acute respiratory syndrome coronavirus 2 (SARS-CoV-2) has become a global epidemic. The clinical manifestations of SARS-CoV-2 infection could range from asymptomatic, cough, fever to dyspnea and even death. As of August 18, 2024, COVID-19 continues to strain healthcare systems worldwide, with exceeding 776 million infections and 7 million deaths reported.^1^ Although vaccination could mitigate the impact of SARS-CoV-2, particularly for high-risk groups, vaccination remains less effective in preventing infections caused by variants with strong immune evasion capabilities.^2,3^ COVID-19 treatment drugs are mainly divided into antiviral drugs and immunomodulatory drugs. Although multiple studies have confirmed the efficacy of immunomodulatory medications, including glucocorticoids, and IL-6 receptor antagonists, the efficacy of immunomodulatory drugs is mostly limited to severe or critical COVID-19 patients.^4,5^ Therefore, the development of effective antiviral drugs is crucial for treating COVID-19 and preventing severe cases and long COVID-19.

Up to now, several medications have been developed for the treatment of COVID-19, such as simnotrelvir, azvudine, molnupiravir, and nirmatrelvir-ritonavir (Paxlovid).^6–10^ In February 2022, Paxlovid received conditional emergency approval in China for the treatment of adult patients with mild-to-moderate COVID-19 who are at high risk for developing severe disease.^11^ Nirmatrelvir, a peptidomimetic inhibitor of SARS-CoV-2 main protease (namely the 3-chymotrypsin-like cysteine protease), can prevent the enzyme from catalyzing the cleavage of viral polyproteins into nonstructural proteins that are elementary for viral replication, thereby inhibiting viral replication.^12^ When included in combination with nirmatrelvir, ritonavir can inhibit the metabolism of nirmatrelvir that was mediated by CYP3A, thus elevating its blood levels. A phase II/III clinical trial for non-hospitalized adult COVID-19 patients at high risk of progressing severe disease proved that Paxlovid could lessen the risk of COVID-19-related hospitalization or death by 89% compared with placebo in patients treated within three days of symptom onset; and reduce the risk of hospitalization or death by 88% in patients treated within five days of symptom onset.^6^ A retrospective cohort study revealed that Paxlovid was associated with a 66% lower risk of death, 34% lower risk of hospitalization, and 43% lower risk of in-hospital disease progression than patients without antiviral drugs.^13^ Several real-world studies have confirmed that Paxlovid effectively reduces disease severity, decreases mortality rates, and significantly accelerates viral clearance during the Omicron variant surge.^14–17^ Azvudine, the first oral small-molecule inhibitor of COVID-19, was originally developed for the treatment of adult HIV-1-infected patients and was conditionally approved in July 2022 for the treatment of adult patients with COVID-19 in China.^18,19^ As a broad-spectrum RNA virus inhibitor, azvudine is metabolized intracellularly into an active 5′-triphosphate metabolite, which specifically targets the RNA-dependent RNA polymerase of SARS-CoV-2, becoming incorporated during viral RNA synthesis and effectively inhibiting viral replication.^20^ Our previous randomized, open-label, controlled clinical trial demonstrated that azvudine may reduce the conversion time of nucleic acid negative.^21^ Notably, another single-arm clinical trial demonstrated that azvudine might have a significant treated effect on COVID-19 patients under compassionate use.^22^ A phase III, randomized, clinical trial for 172 patients with moderate COVID-19 found that azvudine was associated with shorter hospital stays, shorter time to SARS-CoV-2 negative test results, and lower viral loads compared with placebo.^23^

Large-scale SARS-CoV-2 infections have occurred intermittently in China after adjustments in epidemic prevention policies. During the first wave of infection in December 2022, azvudine and Paxlovid were the key antiviral drugs recommended in the published “COVID-19 diagnosis and treatment plan (trial version 9 or version 10)” announced by the National Health Commission of the People’s Republic of China.^24,25^ Although numerous studies have confirmed the efficacy and safety of azvudine or Paxlovid,^26–31^ the results of comparative analyses of the efficacy and safety of azvudine and Paxlovid remain controversial. Some studies for hospitalized COVID-19 patients have demonstrated that azvudine is related to decreased risks of all‐cause death and composite progression versus Paxlovid.^32,33^ Conversely, a multicenter retrospective cohort study revealed no obvious difference in all-cause death or composite disease progression between COVID-19 patients receiving azvudine and those receiving Paxlovid.^34^ Another study indicated that azvudine treatment had similar effectiveness to Paxlovid in reducing composite outcomes and short-term all-cause death in elderly patients with severe SARS-CoV-2 infection.^35^

Considering the urgent need for multicenter retrospective studies with larger sample size and more rigorous statistical methods to clarify the efficacy and safety of azvudine compared with Paxlovid, we conducted this large-scale, multicenter, retrospective, cohort study, which included 40,876 hospitalized patients with SARS-CoV-2 infection from a total of 11 hospitals in Henan and Xinjiang provinces. Furthermore, we performed in vivo and in vitro experiments to verify the potential anti-hepatocellular carcinoma (HCC) effects of azvudine suggested by subgroup analysis, and revealed its potential anti-HCC mechanism through single-cell RNA sequencing. Our results may provide more robust evidence and more novel treatment strategies for the clinical practice of treating SARS-CoV-2 infection.

## Results

### Baseline characteristics

**Fig. 1:**
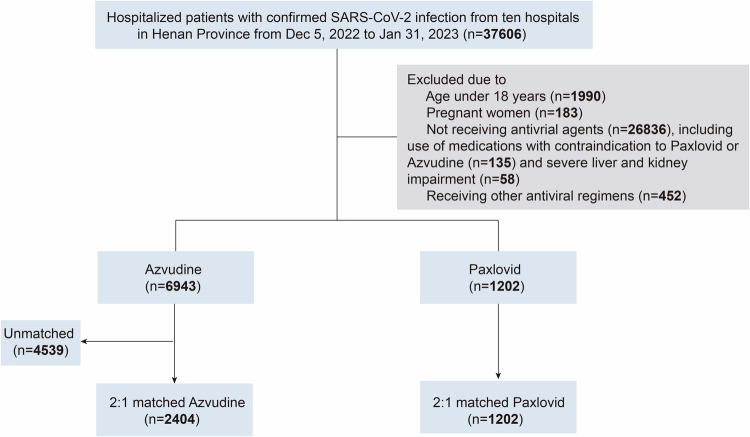
Flowchart of the Henan cohort study design

The information about the demographic and clinical characteristics of a total of 37,606 patients with confirmed COVID-19 were extracted from the electronic medical records of ten hospitals in Henan Province (Fig. 1). Based on the rigorous inclusion and exclusion criteria, 6943 patients who received azvudine and 1202 patients who received Paxlovid were included for further analysis in this study. We subsequently applied 2:1 propensity score matching (PSM) to control for confounders, resulting in the inclusion of 2404 patients in the azvudine group and 1202 patients in the Paxlovid group.

The baseline clinical characteristics of the included patients are shown in Table 1. Before matching, compared with azvudine recipients, Paxlovid recipients were more likely to be male; presented with more severe illness on admission; had fewer concomitant antibiotics and systemic steroids; had longer time from diagnosis to treatment; had fewer kidney diseases; had more liver diseases, cardio-cerebral diseases, and autoimmune diseases; had higher glomerular filtration rate and alkaline phosphatase levels; and had lower neutrophil, prothrombin time, cholesterol, albumin, and total bilirubin levels. After matching, the characteristics of the patients were balanced between azvudine and Paxlovid groups (all *p* > 0.05 and all standardized mean differences (SMD) <0.1) (Supplementary Fig. 1).

**Table 1:** Baseline characteristics of patients with COVID-19 in the Henan cohort before and after propensity score matching

Characteristics	Before matching			After 2:1 matching		
	Azvudine (*n* = 6943)	Paxlovid (*n* = 1202)	*P* value	Azvudine (*n* = 2404)	Paxlovid (*n* = 1202)	*P* value
Age, mean (SD), year	68.37 (15.22)	69.26 (14.81)	0.058	68.88 (14.72)	69.26 (14.81)	0.455
Gender, *n* (%)			<0.001			0.529
Male	4173 (60.1)	790 (65.7)		1553 (64.6)	790 (65.7)	
Female	2770 (39.9)	412 (34.3)		851 (35.4)	412 (34.3)	
BMI, mean (SD), kg/m^2^	24.23 (3.94)	24.44 (3.84)	0.097	24.39 (4.01)	24.44 (3.84)	0.726
Severity at admission, *n* (%)			<0.001			0.724
Mild	977 (14.1)	57 (4.7)		117 (4.9)	57 (4.7)	
Moderate	4459 (64.2)	740 (61.6)		1509 (62.8)	740 (61.6)	
Severe	1507 (21.7)	405 (33.7)		778 (32.4)	405 (33.7)	
Vaccination doses, *n* (%)			0.088			0.902
0 dose	2228 (32.1)	343 (28.5)		690 (28.7)	343 (28.5)	
1 dose	417 (6.0)	74 (6.2)		142 (5.9)	74 (6.2)	
2 doses	921 (13.3)	190 (15.8)		355 (14.8)	190 (15.8)	
3 doses	3313 (47.7)	583 (48.5)		1196 (49.8)	583 (48.5)	
4 doses	62 (0.9)	12 (1.0)		21 (0.9)	12 (1.0)	
5 doses	2 (0.0)	0 (0.0)		0 (0.0)	0 (0.0)	
Time from diagnosis to treatment exposure, *n* (%)			<0.001			0.214
>5 days	1136 (16.4)	409 (34.0)		767 (31.9)	409 (34.0)	
0–5 days	5807 (83.6)	793 (66.0)		1637 (68.1)	793 (66.0)	
Concomitant antibiotics, *n* (%)			<0.001			0.875
No	3070 (44.2)	743 (61.8)		1478 (61.5)	743 (61.8)	
Yes	3873 (55.8)	459 (38.2)		926 (38.5)	459 (38.2)	
Concomitant systemic steroid, *n* (%)			<0.001			0.557
No	3875 (55.8)	795 (66.1)		1566 (65.1)	795 (66.1)	
Yes	3068 (44.2)	407 (33.9)		838 (34.9)	407 (33.9)	
Comorbidities, *n* (%)						
Diabetes	1764 (25.4)	322 (26.8)	0.328	627 (26.1)	322 (26.8)	0.679
Hypertension	3106 (44.7)	503 (41.8)	0.067	987 (41.1)	503 (41.8)	0.676
Liver diseases	770 (11.1)	242 (20.1)	<0.001	443 (18.4)	242 (20.1)	0.236
Cardio-cerebral diseases	2196 (31.6)	434 (36.1)	0.002	817 (34.0)	434 (36.1)	0.221
Kidney diseases	1864 (26.8)	247 (20.5)	<0.001	494 (20.5)	247 (20.5)	1.0
Primary malignant tumor	730 (10.5)	147 (12.2)	0.085	303 (12.6)	147 (12.2)	0.789
Chronic respiratory diseases	1164 (16.8)	180 (15.0)	0.133	356 (14.8)	180 (15.0)	0.934
Autoimmune diseases	234 (3.4)	59 (4.9)	0.01	119 (5.0)	59 (4.9)	1.0
Laboratory parameters, mean (SD)						
Neutrophil, ×10^9^/L	5.81 (4.80)	6.45 (4.74)	<0.001	6.08 (4.24)	6.45 (4.74)	0.017
Lymphocyte, ×10^9^/L	1.20 (4.53)	1.17 (3.52)	0.796	1.14 (2.49)	1.17 (3.52)	0.746
Glucose, mmol/L	8.02 (4.13)	8.23 (4.26)	0.103	8.14 (4.28)	8.23 (4.26)	0.537
High-density lipoprotein, mmol/L	1.16 (2.09)	1.09 (1.30)	0.269	1.07 (1.20)	1.09 (1.30)	0.629
Low-density lipoprotein, mmol/L	2.36 (2.26)	2.28 (1.38)	0.223	2.27 (1.81)	2.28 (1.38)	0.921
Alanine aminotransferase, IU/L	37.51 (101.01)	40.71 (74.34)	0.295	39.45 (80.19)	40.71 (74.34)	0.649
Aspartate aminotransferase, IU/L	42.83 (153.92)	39.69 (52.09)	0.485	39.89 (68.93)	39.69 (52.09)	0.93
Creatine, μmol/L	108.00 (180.11)	97.70 (132.22)	0.058	99.88 (158.58)	97.70 (132.22)	0.68
Glomerular filtration rate, ml/min	80.47 (55.31)	84.34 (60.65)	0.027	84.33 (73.32)	84.34 (60.65)	0.998
C-reactive protein, mg/L	53.98 (65.79)	57.48 (64.84)	0.088	57.97 (70.25)	57.48 (64.84)	0.842
Procalcitonin, ng/ml	1.24 (7.22)	1.25 (6.67)	0.954	1.18 (7.01)	1.25 (6.67)	0.762
Prothrombin time, s	16.95 (10.29)	15.48 (10.47)	<0.001	15.44 (8.22)	15.48 (10.47)	0.899
Activated partial thromboplastin time, s	27.54 (12.18)	28.22 (15.28)	0.084	28.02 (10.45)	28.22 (15.28)	0.639
Cholesterol, mmol/L	4.01 (2.28)	3.85 (1.13)	0.022	3.85 (1.58)	3.85 (1.13)	0.992
Triglyceride, mmol/L	1.50 (2.42)	1.41 (1.43)	0.181	1.40 (1.51)	1.41 (1.43)	0.958
Alkaline phosphatase, IU/L	82.46 (52.57)	87.71 (92.21)	0.005	85.82 (62.85)	87.71 (92.21)	0.47
Gamma-glutamyl transpeptidase, IU/L	58.03 (125.51)	59.65 (71.84)	0.664	61.03 (93.80)	59.65 (71.84)	0.654
Albumin, g/L	36.76 (34.00)	33.57 (8.77)	0.001	33.56 (13.52)	33.57 (8.77)	0.976
Total bilirubin, μmol/L	12.19 (11.29)	11.44 (12.31)	0.037	11.42 (10.05)	11.44 (12.31)	0.963

### Efficacy of azvudine versus Paxlovid on all-cause death and composite disease progression

**Fig. 2:**
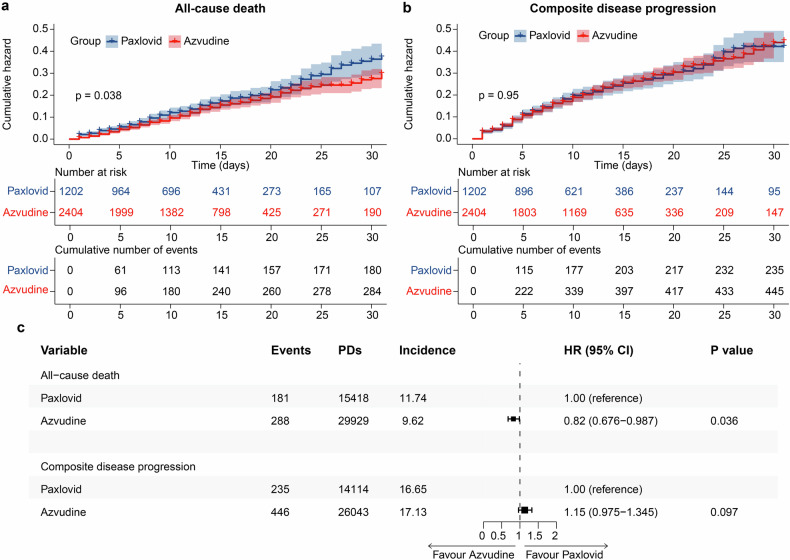
Kaplan–Meier curves and multivariate Cox proportional hazards regression analysis of patients receiving azvudine treatment versus Paxlovid treatment in the Henan cohort. Cumulative hazard of all‐cause death (**a**) and composite disease progression (**b**) assessed by Kaplan–Meier curves. **c** Hazard ratio of all-cause death and composite disease progression after adjusting for all baseline covariates in Table 1. HR hazard ratio, 95% CI 95% confidence interval, PDs Person-days, Incidence events/per 1000 PDs

In the Henan cohort, there were 469 all-cause deaths, including 288 in the azvudine group and 181 in the Paxlovid group. Compared with Paxlovid treatment, azvudine therapy was related to an obviously lower risk of all-cause death, as determined by the log-rank test (*p* = 0.038) (Fig. 2a). After multivariable adjustment using Cox regression analysis, the hazard ratio (HR) for all-cause death in the azvudine group was 0.82 (95% confidence intervals (CI): 0.676–0.987, *p* = 0.036) versus that in the Paxlovid group (Fig. 2c).

The secondary outcome was composite disease progression. A total of 681 secondary outcome events occurred, including 446 in the azvudine group and 235 in the Paxlovid group. The cumulative hazard of composite disease progression was not significantly different between the azvudine and Paxlovid treatments (*p* = 0.95) (Fig. 2b). Cox analysis revealed that after adjusting the confounding factors, the HR was 1.15 (95% CI: 0.975-1.345, *p* = 0.097) in the azvudine group compared with the Paxlovid group (Fig. 2c).

### Sensitivity analyses and cross-region cohort validation

The dependability of clinical research results could be influenced by various factors, including data quality, the analysis population, and statistical methods. After the initial results were obtained, we speculated that different approaches to handling missing data, varying matching models, or different analyzing populations might have affected our findings. Therefore, we conducted the following three sensitivity analyses for the Henan cohort to demonstrate the robustness of our conclusions.

First, when missing values were addressed via mean imputation (Supplementary Table 1), Kaplan–Meier analysis revealed there was no notable difference in the risk of all-cause death between azvudine and Paxlovid groups (*p* = 0.053) (Supplementary Fig. 2a). However, Cox regression analysis indicated that patients receiving azvudine had a 21% lower risk of all-cause death than did those with Paxlovid treatment (95% CI: 0.658–0.959, *p* = 0.016) (Supplementary Fig. 2c). The risk of composite disease progression was consistent with results obtained from the original dataset (Kaplan–Meier analysis: *p* = 0.78; Cox analysis: HR: 1.11, 95% CI: 0.944–1.299, *p* = 0.209) (Supplementary Fig. 2b, c).

Second, when a Probit model was used to perform a 1:2 greedy match (Supplementary Table 2), Kaplan–Meier analysis (*p* = 0.0041) (Supplementary Fig. 3a) and Cox regression analysis (HR: 0.73, 95% CI: 0.603–0.884, *p* = 0.001) (Supplementary Fig. 3c) indicated that azvudine group was related to a lower risk of all-cause death than that in the Paxlovid group. The risk of composite disease progression was not obviously different between the two groups (Kaplan–Meier analysis: *p* = 0.91; Cox analysis: HR: 1.11, 95% CI: 0.947–1.305, *p* = 0.194) (Supplementary Fig. 3b, c).

Third, the results remained robust when the analysis was repeated after patients discharged from the hospital on the first day after admission were excluded (Supplementary Table 3). In this analysis, azvudine was related to the decreased risk of all-cause death versus Paxlovid, as demonstrated by Kaplan–Meier analysis (*p* = 0.031) (Supplementary Fig. 4a) and Cox analysis (HR: 0.80, 95% CI: 0.657–0.964, *p* = 0.02) (Supplementary Fig. 4c). Furthermore, no obvious difference in cumulative incidence of composite disease progression was observed between two groups (Kaplan–Meier analysis: *p* = 0.8; Cox analysis: HR: 1.13, 95% CI: 0.960–1.335, *p* = 0.139) (Supplementary Fig. 4b, c).

In addition to sensitivity analysis, to demonstrate the generalizability of our results, we collected 3270 hospitalized COVID-19 patients from outside the Henan region of Xinjiang Province. After exclusion and PSM, a total of 79 azvudine recipients and 78 Paxlovid recipients were enrolled (Supplementary Fig. 5) (Supplementary Table 4). Kaplan–Meier analysis suggested no notable difference in the risks of all-cause mortality (*p* = 0.39) or composite disease progression (*p* = 0.27) between the two groups (Supplementary Fig. 6a, b). Cox regression analysis indicated that the azvudine group had a lower risk of all-cause death than the Paxlovid group did (HR: 0.53, 95% CI: 0.283–0.989, *p* = 0.046), and with no obvious difference in the risk of composite disease progression (HR: 0.57, 95% CI: 0.322–1.021, *p* = 0.059) (Supplementary Fig. 6c).

### Safety

We collected data on adverse events (AEs) during follow-up for both the azvudine and Paxlovid groups (Supplementary Table 5). Compared with azvudine recipients, patients in the Paxlovid group had a greater risk of Grade 1 AEs, including increased alanine aminotransferase (ALT) (*p* = 0.013), hypercholesterolemia (*p* < 0.001), and increased aspartate aminotransferase (AST) (*p* = 0.047). With respect to Grade 2 AEs, Paxlovid administration was related to greater risks of increased decreased platelet (*p* = 0.009), increased creatinine (*p* = 0.018), and ALT (*p* = 0.036) than azvudine. For Grade 3 and greater SEs, Paxlovid treatment was related to a higher incidence of decreased lymphocyte count (*p* < 0.001).

### Survival prediction model

**Fig. 3:**
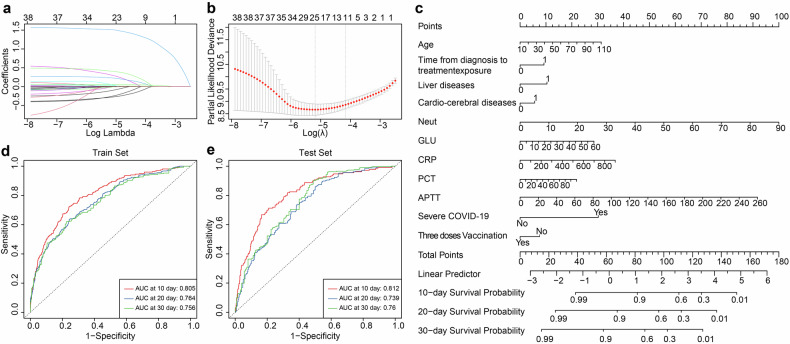
Nomogram for survival prediction of hospitalized COVID-19 patients receiving azvudine in the Henan cohort. **a** LASSO coefficient profile of 40 features. **b** Tuning parameter (λ) selection in the LASSO model via fivefold cross-validation. **c** Nomogram to estimate 10-, 20-, and 30-day survival for COVID-19 patients receiving azvudine. The prediction performance of the LASSO model evaluated by ROC curves in the training set (**d**) and test set (**e**)

The LASSO regression analysis included thirty-eight variables measured at hospital admission (Fig. 3a). The optimal λ value, λ.1 se, was chosen on the basis of fivefold cross-validation (Fig. 3b). Eleven features were identified and used to establish a nomogram for predicting 10-, 20-, and 30-day survival probabilities in patients receiving azvudine (Fig. 3c). In the training set, the area under the receiver operating characteristic curve (AUCs) of our model were 80.5%, 76.4%, and 75.6% for 10-, 20-, and 30-day survival, respectively (Fig. 3d); in the test set, they were 81.2%, 73.9%, and 76%, respectively (Fig. 3e); and in the external validation set, they were 78.3%, 70.3%, and 90.4%, respectively (Supplementary Fig. 7a). For patients receiving Paxlovid, an additional nomogram was constructed to predict the 10-, 20-, and 30-day survival probabilities (Supplementary Fig. 8a–c). In the training set, the AUCs of the model were 80.7%%, 83.2%, and 86.1% for 10-, 20-, and 30-day survival, respectively (Supplementary Fig. 8d); in the test set, they were 80.1%%, 81.1%, and 87.8%, respectively (Supplementary Fig. 8e); and in the external validation set, they were 65.7%, 51.9%, and 68.2% (Supplementary Fig. 7b).

The concordance index (C-index) of survival prediction model for azvudine recipients was 0.821 in the training set, 0.819 in the test set (Supplementary Fig. 9a, b), and 0.833 in the cross-region validation cohort (Supplementary Fig. 7c). In addition, the C-index for the Paxlovid nomogram was 0.793 in the training set, 0.792 in the test set (Supplementary Fig. 9c, d), and 0.703 in the external validation set (Supplementary Fig. 7d). The calibration curves for both nomograms proved great consistency between the predicted and observed survival probabilities in the training and test sets. The decision curve analysis (DCA) curves indicated that using the nomogram to predict survival in COVID-19 patients receiving azvudine or Paxlovid provided greater net benefit than either predicting all patients as survivors or predicting none as survivors across almost all threshold probabilities in both the training and test sets (Supplementary Fig. 9e–h).

### Subgroup analysis

To further investigate the effects of antiviral drugs on clinical outcomes across different populations, we stratified the analysis on the basis of sex, age, severity, vaccination dose, concomitant antibiotic use, systemic steroid use, time from diagnosis to treatment exposure, and comorbidities.

For all-cause death, potentially meaningful interactions suggesting a greater benefit of azvudine over Paxlovid were observed in patients who started treatment >5 days after diagnosis (*p* for interaction = 0.009, HR = 0.56, 95% CI: 0.39−0.78), in those with primary malignant tumors (*p* for interaction <0.001, HR: 0.33, 95% CI: 0.20−0.54), and in those without systemic steroid use (*p* for interaction = 0.004, HR: 0.67, 95% CI: 0.53‒0.84) (Table 2). For composite disease progression, potentially meaningful interactions suggesting a greater benefit of azvudine over Paxlovid were observed in patients with moderate COVID-19 (*p* for interaction = 0.036, HR: 0.67, 95% CI: 0.45−1.00) and with primary malignant tumors (*p* for interaction = 0.012, HR: 0.54, 95% CI: 0.33−0.88) (Table 2).

**Table 2:** Subgroup analyses in the Henan cohort for the effects of different drug treatments on all-cause death and composite outcomes according to baseline characteristics

Characteristic	Count	Percent	All-cause death		Composite disease progression	
			HR (95%CI)^a^	*P* value for interaction	HR (95%CI)^a^	*P* value for interaction
Age, year						
≤60 year	890	24.7	0.86 (0.55−1.36)	**0.853**	0.92 (0.63−1.35)	**0.579**
>60 year	2716	75.3	0.81 (0.66−0.99)		1.02 (0.86−1.21)	
Gender						
Male	2343	65	0.85 (0.68–1.05)	**0.536**	1.04 (0.87−1.26)	**0.396**
Female	1263	35	0.75 (0.53–1.08)		0.88 (0.65−1.20)	
Severity at admission						
Mild	174	4.8	0.76 (0.20−2.82)	**0.235**	1.09 (0.32−3.72)	**0.036**
Moderate	2249	62.4	0.60 (0.40−0.90)		0.67 (0.45−1.00)	
Severity	1183	32.8	0.89 (0.72−1.10)		1.17 (0.98−1.39)	
Vaccination doses						
None	1033	28.6	0.95 (0.70−1.28)	**0.258**	1.13 (0.86−1.47)	**0.109**
One dose	216	6.0	0.51 (0.28−0.95)		0.66 (0.38−1.12)	
Two doses	545	15.1	0.76 (0.45−1.29)		1.19 (0.77−1.86)	
Three doses	1779	49.3	0.79 (0.59−1.07)		0.92 (0.72−1.17)	
Four doses	33	0.9	0.00 (0.00−Inf)		0.00 (0.00−Inf)	
Concomitant Antibiotics						
No	2221	61.6	0.75 (0.57−0.97)	**0.233**	1.02 (0.81−1.27)	**0.999**
Yes	1385	38.4	0.92 (0.71−1.20)		1.01 (0.81−1.27)	
Concomitant systemic steroid, *n* (%)						
No	2361	65.5	0.67 (0.53−0.84)	**0.004**	0.92 (0.75−1.12)	**0.138**
Yes	1245	38.4	1.19 (0.86−1.64)		1.15 (0.89−1.48)	
Time from diagnosis to treatment exposure						
>5 days	1176	32.6	0.56 (0.39−0.78)	**0.009**	0.93 (0.70−1.23)	**0.494**
≤5 days	2430	67.4	0.96 (0.76−1.20)		1.04 (0.86−1.26)	
Diabetes, n (%)						
No	2657	73.7	0.83 (0.67−1.04)	**0.839**	0.99 (0.82−1.19)	**0.743**
Yes	949	26.3	0.80 (0.56−1.13)		1.05 (0.78−1.42)	
Hypertension, *n* (%)						
No	2116	58.7	0.73 (0.57−0.93)	**0.13**	0.85 (0.69−1.04)	**0.016**
Yes	1490	41.3	0.98 (0.73−1.31)		1.27 (0.99−1.62)	
Liver diseases, n (%)						
No	2921	81	0.74 (0.59−0.92)	**0.06**	0.99 (0.82−1.18)	**0.547**
Yes	685	19	1.09 (0.77−1.54)		1.09 (0.80−1.49)	
Cardio-cerebral diseases, *n* (%)						
No	2355	65.3	0.83 (0.64−1.07)	**0.936**	0.99 (0.80−1.21)	**0.685**
Yes	1251	34.7	0.83 (0.63−1.09)		1.06 (0.83−1.35)	
Kidney diseases, *n* (%)						
No	2865	79.5	0.82 (0.66−1.03)	**0.919**	1.03 (0.85−1.25)	**0.732**
Yes	1251	20.5	0.83 (0.59−1.17)		0.98 (0.74−1.28)	
Primary malignant tumor, *n* (%)						
No	3156	87.5	0.94 (0.77−1.16)	**<0.001**	1.08 (0.91−1.27)	**0.012**
Yes	450	12.5	0.33 (0.20−0.54)		0.54 (0.33−0.88)	
Chronic respiratory diseases						
No	3070	85.1	0.86 (0.71−1.05)	**0.124**	1.06 (0.90−1.26)	**0.07**
Yes	536	14.9	0.54 (0.31−0.95)		0.69 (0.45−1.06)	
Autoimmune diseases						
No	3428	95.1	0.82 (0.68−0.99)	**0.943**	1.02 (0.87−1.20)	**0.34**
Yes	178	4.9	0.78 (0.32−1.90)		0.72 (0.35−1.49)	

*HR* hazard ratio, *95% CI* 95% confidence interval

^a^The ratio of the probability of an outcome event in the azvudine group to the probability in the Paxlovid group

### Antitumor effects of azvudine in vivo and in vitro

**Fig. 4:**
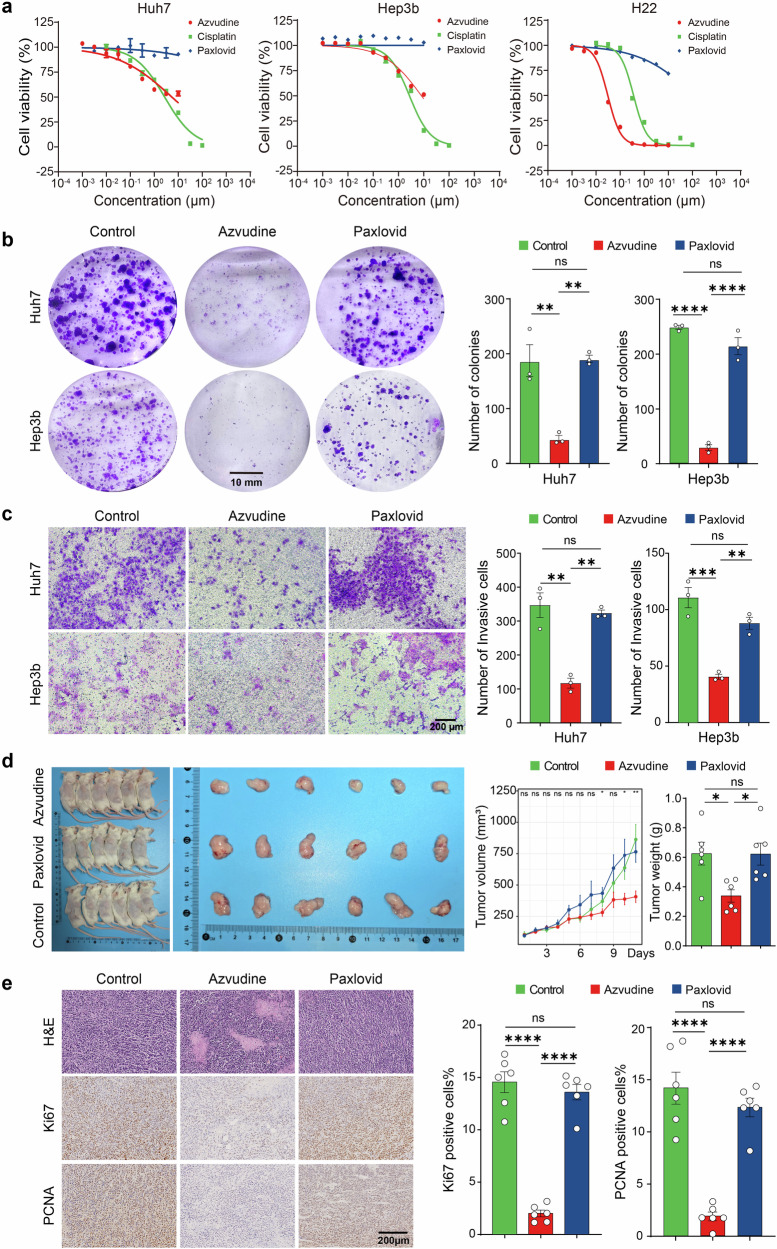
Azvudine inhibits tumor growth in vitro and in vivo. **a** The proliferation of Huh7, Hep3b, and H22 cells treated with azvudine, Paxlovid, and cisplatin was assessed via the CTG assay. **b** The proliferation of Hep3b and Huh7 cells treated with azvudine, Paxlovid, or DMSO was assessed by a colony formation assay (*n* = 3 per group). Scar bar, 10 mm. **c** The invasion of Hep3b and Huh7 cells treated with azvudine, Paxlovid, or DMSO was evaluated by Transwell assays (*n* = 3 per group). *p < 0.05, **p < 0.01, ***p < 0.001, ****p < 0.0001. Scar bar, 200 μm. **d** Tumor growth and weight were monitored in BALB/c mice subcutaneously injected with H22 cells (*n* = 6 per group). **e** H&E and IHC staining of Ki-67 and PCNA expression in tumors (*n* = 6 per group). The error bars represent the means ± SEs. Scar bar, 200 μm

Subgroup analysis indicated that azvudine had a more pronounced benefit in reducing clinical outcomes in patients with primary malignant tumors. To further explore the antitumor role of azvudine, we investigated its role in HCC cell lines (H22, Hep3b, and Huh7) and lung cancer (LC) cell lines (NCI-H1975 and NCI-H82). Interestingly, CTG assays revealed that azvudine obviously inhibited the proliferation of all the tested cell lines (Fig. 4a and Supplementary Fig. 10). In contrast, no notable difference was observed in the Paxlovid versus the control. Similarly, colony formation assays demonstrated that azvudine markedly decreased the number of colonies (Fig. 4b). Additionally, invasion assays revealed that, compared with Paxlovid or DMSO treatment, azvudine treatment significantly reduced the number of invading HCC cells (Fig. 4c). These results suggest that azvudine can effectively inhibit cancer cell proliferation and invasion.

To further evaluate the antitumor effects of azvudine in vivo, an allograft mouse model was generated by subcutaneously injecting H22 cells into mice. The mice were then randomly assigned into three groups: the azvudine group (1 mg/kg, once daily), the Paxlovid group (61.65 mg/kg nirmatrelvir and 20.55 mg/kg ritonavir, twice daily), and the control group (solvent). In line with the in vitro results, tumor growth rate and average tumor weight in the azvudine group were obviously lower than those in the control, and Paxlovid groups (Fig. 4d). Immunohistochemistry (IHC) analysis revealed azvudine group had a weaker Ki-67 and PCNA staining than in the Paxlovid and control groups (Fig. 4e). With respect to the biosafety of azvudine and Paxlovid, blood biochemical indicators of liver and kidney function in mice were within the normal range across all three groups (Supplementary Fig. 11a). Additionally, H&E staining of the heart, lungs, spleen, liver, and kidneys further confirmed the safety of both agents (Supplementary Fig. 11b). These results validated the antitumor effects of azvudine in HCC.

### Single-cell RNA sequencing (scRNA-seq) analysis of the tumor immune microenvironment after azvduine treatment

**Fig. 5:**
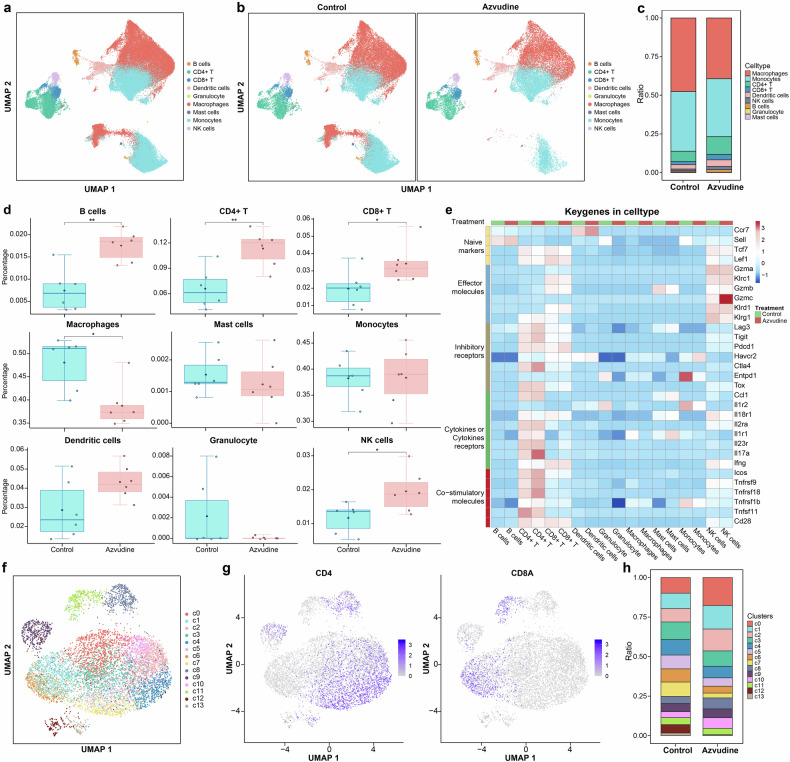
scRNA-seq revealed that the global tumor immune profiles respond to azvudine treatment. **a** UMAP plot of the cluster analysis of all samples, with each color denoting a distinct cell type. **b** Distribution of nine immune cell types in the control and azvudine groups (*n* = 6 per group). **c** Proportion of immune cell types in the control and azvudine groups (*n* = 6 per group). **d** Frequencies of different immune cell types in the control and azvudine groups. *p < 0.05, **p < 0.01, ***p < 0.001. **e** The expression levels of characteristic genes across nine immune cell types in the control and azvudine groups. **f** UMAP plot of T lymphocyte subclusters in all samples. **g** Expression profiles of specific marker genes for T cell subclusters in UMAP. **h** Proportion of T cell subclusters in the control and azvudine groups. C cluster

We subsequently performed scRNA-seq on mouse HCC tumor tissue to explore the potential mechanism of the antitumor effect of azvudine. Nine distinct immune cell types were recognized by uniform manifold approximation and projection (UMAP) analysis (Fig. 5a). The distribution of immune cell types in the control group differed from that in the azvudine group (Fig. 5b). Macrophages, monocytes, and CD4^+^ T cells composed the dominant immune cell types in the two groups (Fig. 5c). The proportions of CD4^+^ T cells, CD8^+^ T cells, B cells, and NK cells were significantly greater in the azvudine group than in the control group (Fig. 5d). Conversely, the proportion of macrophages was decreased in the azvudine group. We analyzed the expression levels of typical molecular markers in both groups to investigate the effect of azvudine on functional alterations in immune cells (Fig. 5e). Following treatment with azvudine, the costimulatory molecules ICOS and TNFRSF9 were upregulated in CD4^+^ T cells, whereas Tnfrsf11 was downregulated in CD4^+^ T cells; the cytokine receptor IL17a was upregulated in CD4^+^ T cells; IL1R1 was upregulated in mast cells; and the inhibitory receptor ENTPD1 was downregulated in granulocytes and monocytes.

To identify the phenotypic heterogeneity of T cells, UMAP analysis were performed on the scRNA-seq data from T cells (Fig. 5f–h). A total of 14T lymphocyte clusters were recognized. Compared with those in the control group, the proportions of subclusters 0 (MT2^+^CD4^+^ T cells) and 2 (IKZF2^+^CD4^+^ T cells) were notably greater in azvudine group, while those of subclusters 5 (IL18RAP^+^CD4^+^ T cells), 6 (LY6C2^+^CD8^+^ T cells), and 7 (CXCR6^+^CD4^+^ T cells) were significantly lower (Supplementary Fig. 12a). Following azvudine treatment, the effector molecule KLRG1 was downregulated in subcluster 2, GZMC was upregulated in subcluster 13 (Supplementary Fig. 12b), the inhibitory receptor TIGIT was upregulated in subcluster 12, and the costimulatory molecule CCL1 was upregulated in subclusters 4 and 7.

**Fig. 6:**
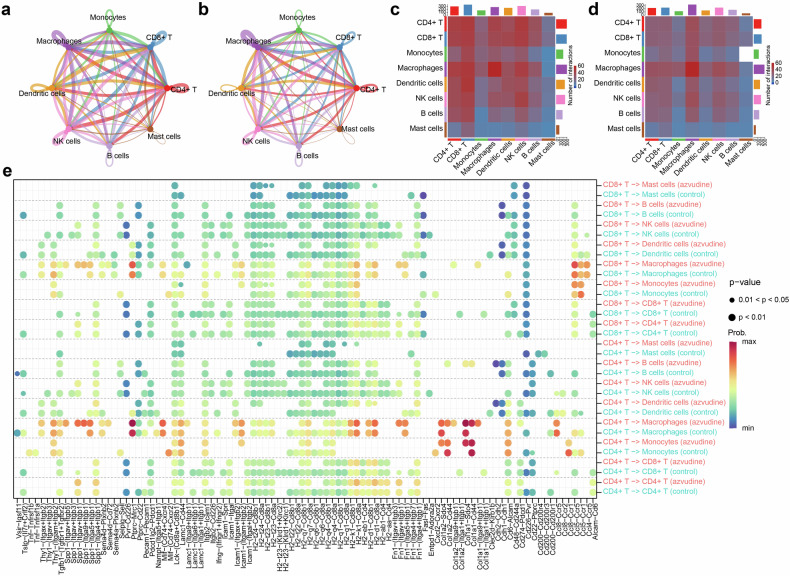
Azvudine reduces cell–cell interactions and communication among immune cells in HCC. The number of intercellular interactions in the control (**a**) and azvudine groups (**b**). Heatmap displaying the numbers of cell–cell interactions in the control (**c**) and azvudine (**d**) groups. **e** Bubble map presenting the comparison of significant ligand–receptor pairs in the control and azvudine groups when CD4^+^ T and CD8^+^ T cells were used as ligand cells

The ligand‒receptor interactions across diverse immune cell types were analyzed via CellChat (Fig. 6a, b). The total interaction strength and intercellular interaction number were lower in the azvudine group than in the controls (Fig. 6c, d and Supplementary Fig. 12c). Ligand‒receptor analysis revealed that azvudine predominantly regulated signal transduction from tumor CD4^+^ T cells to other immune cells through SPP1-(ITGAV + ITGB5) and SPP1-(ITGAV + ITGB1), as well as COL1A1-(ITGA9 + ITGB1), COL1A1-CD44, and COL1A1-SDC4 (Fig. 6e). Azvudine mainly mediated intercellular interactions from CD8^+^ T cells to other immune cells through influencing SPP1-(ITGAV + ITGB1), SPP1-(ITGA9 + ITGB1), and SPP1-(ITGA5 + ITGB1). Additionally, azvudine regulated the interactions from other tumor immune cells to CD4^+^ T and CD8^+^ T cells through ligand‒receptor pairs such as COL1A2‒(ITGA1 + ITGB1), CCL4‒CCR5, CCL6‒CCR2, and CXCL16‒CXCR6 (Supplementary Fig. 13).

The pseudotime trajectories of the CD4^+^ and CD8^+^ T cell subclusters between two groups were obtained via the Monocle algorithm (Supplementary Fig. 14a, d). The results revealed that azvudine may influence the development and differentiation of CD4^+^ and CD8^+^ T cells (Supplementary Fig. 14b, e). We observed that subclusters 4, 5, 6, and 7 were comprised primarily of cells in the initial stage of development that may differentiate into subclusters 0, 9, 11, 12, and 13 (Supplementary Fig. 14c, f). KEGG enrichment analysis suggested that the differentially expressed genes (DEGs) in CD4^+^ T cells were involved mainly in cytokine‒cytokine receptor interactions and prion disease (Supplementary Fig. 15a, b), whereas DEGs in CD8^+^ T cells were involved primarily in protein processing in endoplasmic reticulum and cytokine‒cytokine receptor interactions (Supplementary Fig. 15c, d).

## Discussion

Given the limitations of previous studies, including single-center designs, small sample sizes, and insufficient analytical rigor, there is an urgent need for a large-scale, multicenter, retrospective, cohort study to compare the efficacy and safety of azvudine and Paxlovid drugs in hospitalized patients with COVID-19. Therefore, we conducted this largest multicenter, real-world study. In this study, we enrolled 37,606 hospitalized patients with COVID-19 from ten hospitals in Henan Province, China, ultimately including 2404 patients treated with azvudine and 1202 treated with Paxlovid. Notably, we included patient vaccination information, which was largely absent from previous studies but is crucial for understanding disease progression and clinical outcomes.^36^ In our cohort, the vaccination coverage rate of the hospitalized population was 68.43%, with unvaccinated patients accounting for 31.57% of the population. Our results revealed that patients receiving azvudine had a lower risk of all-cause death than those receiving Paxlovid, with no obvious difference in composite disease progression. This consistent effect was observed across subgroups stratified by age, sex, vaccination dose, concomitant antibiotics, and various comorbidities, including chronic respiratory diseases, diabetes, liver disease, kidney disease, cardio-cerebral disease, and autoimmune diseases. Notably, azvudine therapy significantly reduced the risks of all-cause death and/or composite disease progression compared with Paxlovid therapy in patients with >5 days from diagnosis to treatment, those with moderate COVID-19, those not receiving systemic steroids, and particularly in patients with primary malignant tumors. Additionally, we included COVID-19 patients from Xinjiang Province and employed various method for handling missing data, different matching model, and different analysis population to reanalyze the data, consistently yielding the same results. This robustness further demonstrates the reliability of our conclusions.

Guidelines from the WHO and China recommend Paxlovid for patients with mild‐to‐moderate COVID‐19 who are at high risk for hospitalization,^37^ whereas azvudine is recommended for moderate cases.^25^ However, under the first Omicron infection wave in China, many COVID-19 patients in real-world settings took these drugs off-label. This study included patients with mild to severe COVID-19 and found that the effect of azvudine on all-cause death was consistent across all severity subgroups. Compared with Paxlovid therapy, azvudine therapy was related to a 33% lower risk of composite disease progression in moderate COVID-19. In addition, owing to the vulnerability of elderly individuals, who are more susceptible to severe illness and hospitalization after SARS-CoV-2 infection,^38,39^ the study population was older, with an average age of 68.50 years. Our findings demonstrate that the effects of azvudine on clinical outcomes were consistent across all age subgroups. These results provide additional options for clinical treatment.

The results of subgroup analyses suggested that azvudine was superior to Paxlovid in reducing clinical outcomes in patients with malignant tumors. Intriguingly, further cell and animal experiments revealed that azvudine could inhibit the proliferation and invasion ability of HCC cells and suppress the growth of allograft HCC tumors in vivo, whereas Paxlovid did not exhibit any antitumor effects. The scRNA-seq analysis demonstrated a notable raise in the proportions of CD4^+^ T and CD8^+^ T cells after azvudine treatment. The expression of the functional markers TNFRSF9, IL17a, and ICOS in CD4^+^ T cells was significantly upregulated, suggesting that the regulatory function of CD4^+^ T cells was altered. Our discoveries provide valuable insights into the use of azvudine in COVID-19 patients with tumors in clinical practice. However, the antitumor mechanism and expanded use of azvudine require further validation through additional experiments and larger clinical trials to benefit a broader population.

We speculate on the reasons for the unique antitumor effects of azvudine. First, previous studies have demonstrated that azvudine exhibits obvious thymus (lymph) homing characteristics after administration, and its active triphosphate form is gathered in the thymus (lymph) and peripheral blood mononuclear cells, enabling azvudine to activate the immune system while clearing the virus from the peripheral blood. The slow release of active metabolites in the thymus (lymph) allows for continuous viral clearance, thereby protecting the immune system.^22,40,41^ The above studies support our conclusion that azvudine can modulate tumor immunity. Second, ritonavir, a component of Paxlovid, is a CYP3A inhibitor that can impair the detoxification and metabolism of many drugs. Therefore, Paxlovid may interfere with ongoing antitumor treatment and potentially exacerbate existing conditions.^42,43^ Third, the duration of treatment differs between the two drugs. The guidelines from China recommend a 14-day treatment course for azvudine and a 5-day course for Paxlovid.^25^ For patients with tumors, who often have weakened immune defenses against viruses, a viral infection can worsen their underlying disease or even lead to death. A longer antiviral treatment course with azvudine may contribute to better clinical outcomes for cancer patients with COVID-19.

Most previous studies controlled for only confounding factors such as demographic characteristics, medication use, comorbidities, and disease severity, but few studies have considered the effects of laboratory testing indicators.^30,33^ However, studies have demonstrated that neutrophil, monocyte, and lymphocyte counts at admission are related to mortality in COVID-19 patients.^44,45^ Additionally, albumin and creatinine levels can predict acute kidney injury in COVID-19 patients,^46^ and CRP has been identified as an early predictive marker of hypoxia in COVID-19 patients.^47^ These results suggest that many laboratory test indicators could also be potential confounding factors. Therefore, our study controlled for demographic characteristics, laboratory test indicators, and medication use, etc.

There are several limitations in our study. First, although we additionally included patients from outside Henan Province, the sample size was small, which may restrict the generalizability of our findings. Second, we conducted only a short-term follow-up of 31 days and did not evaluate the long-term effectiveness of either drug. Third, despite adjusting for various potential confounders, we could not eliminate the possibility of selection bias or other biases in this retrospective study, such as differences in economic income, clinician treatment preferences, or patient choice. Fourthly, since we were unable to obtain information such as symptoms and signs of hospitalized patients, the AEs in our research only involved abnormal laboratory test results, resulting in an incomplete safety evaluation. Finally, the SARS-CoV-2 virus continues to evolve, and it remains uncertain whether azvudine could be effective against other emerging strains.

In conclusion, this large-scale, multicenter, retrospective, cohort study demonstrated that azvudine is not inferior to Paxlovid in treating hospitalized COVID-19 patients and has few adverse events. Notably, azvudine may offer greater clinical benefits for patients with HCC.

## Materials and methods

### Study population and data sources

We did this multicenter, retrospective, cohort study in Henan Province and Xinjiang Province, China, involving hospitalized patients with confirmed SARS-CoV-2 infection, as detected by reverse transcription polymerase chain reaction (RT‒PCR) between December 5, 2022, and January 31, 2023. This research consisted of two cohorts, one with a total of 37,606 patients from 10 hospitals in Henan Province, and the other with 3270 patients from one hospital in Xinjiang Province (Supplementary Method).

The detailed inclusion criteria of hospitalized patients with COVID-19 were as follows: (1) had a positive RT‒PCR test for SARS-CoV-2; (2) aged 18 years or older; (3) received standard therapy plus either azvudine or Paxlovid. The exclusion criteria were as follows: (1) patients who did not receive any antiviral agents; (2) patients who received other antiviral regimens; (3) pregnant patients; and (4) patients with contraindications to Paxlovid (e.g., severe liver or kidney impairment or the use of medications highly dependent on CYP3A for clearance) or azvudine. All patients received diagnosis and treatment in accordance with the “COVID-19 diagnosis and treatment plan (Trial Version 9 or Version 10)” issued by the National Health Commission of the People’s Republic of China.^24,25^

We retrieved electronic medical records from eleven hospitals and collected data such as demographic characteristics, dates of admissions, intensive care unit admissions, registered deaths, diagnoses, prescription records, imaging data, and laboratory tests.

### Procedures

Eligible participants were allocated to either Paxlovid or azvudine groups based on their drug prescription records. The index date was defined as confirmation of SARS-CoV-2 infection. The Paxlovid and azvudine groups were matched using PSM at a 1:2 ratio. Patients were observed from the index date until clinical outcome events or for 31 days.

The research ethics board at the First Affiliated Hospital of Zhengzhou University approved this study under approval numbers 2023-KY-0865-001 and 2024-KY-1721-001. The research protocol adhered to the STROBE guidelines and the ethical standards outlined in the 1975 Declaration of Helsinki. Since all patients were anonymous, individual informed consent was not required.

### Outcomes

The primary outcome of this study was all-cause death, which was determined on the basis of medical records. The secondary outcome was composite disease progression. Disease progression was defined as progression from mild or moderate disease to severe disease or death.

The safety outcome was evaluated on the basis of AEs classified as Grade 1, Grade 2, or Grade ≥ 3, according to the Common Terminology Criteria for Adverse Events, Version 5.0 (CTCAE 5.0).^48^ The AEs monitored in our research were abnormal laboratory results. The safety evaluation began after the initiation of drug use, with follow-up continuing until five half-lives after the last administration. If the severity of an adverse event changed during follow-up, the highest grade observed was recorded.

### Definition of covariates

Age, sex, and body mass index (BMI) data were obtained from the patients’ demographic records. The severity of patients at admission was classified as “mild”, “moderate”, or “severe” according to the “COVID-19 diagnosis and treatment plan (Trial Version 9 or Version 10)”.^24,25^ Mild cases were defined by the presence of only representative respiratory tract infection symptoms. Moderate COVID-19 were characterized by a continuous high fever lasting more than 3 days, a respiratory rate less than 30 breaths per minute, or an oxygen saturation above 93%, along with characteristic COVID-19 findings on imaging. Severe disease was defined by any of the following criteria: a respiratory rate of 30 or more breaths per minute; resting oxygen saturation of 93% or less; a PaO_2_/FiO_2_ ratio of 300 mmHg or less; lung lesions progressing more than 50% within 24–48 h; mechanical ventilation; shock; intensive care unit monitoring. Concomitant use of systemic steroids or antibiotics was categorized as “No” or “Yes,” depending on whether these treatments were administered within one day of admission. The time from diagnosis to treatment exposure was categorized as “0–5 days” or “>5 days” on the basis of when azvudine and Paxlovid were first administered. The details of laboratory results collected at diagnosis was presented at Supplementary Method.

### Prediction model construction

A total of 6943 azvudine recipients and 1202 Paxlovid recipients from the Henan cohort were randomly divided into training and test sets at a 2:1 ratio, respectively. Moreover, 79 azvudine recipients and 94 Paxlovid recipients from the Xinjiang cohort were used as an external validation set. Potential clinical features were selected using the LASSO regression in the training set. The optimal value of λ was determined as λ.1 se through fivefold cross-validation. The selected features were then entered into a Cox regression model to identify the most predictive factor. A nomogram was constructed on the basis of the final Cox model.

We assessed the model’s performance accuracy using the AUC and C-index. Calibration ability was assessed via calibration curves with 1000 bootstrap resamples. The net benefit threshold for prediction was determined through DCA. Finally, the efficacy of model was validated in the test and external validation sets.

### Cell culture and treatment

The HCC cell lines (Huh7 and Hep3b) and LC cell lines (NCI-H1975 and NCI-H82) were maintained by our laboratory. Huh7 and Hep3b cells were cultured in Dulbecco’s modified Eagle’s medium (high glucose; Gibco, Waltham, USA) supplemented with 10% fetal bovine serum (FBS; VivaCell, Shanghai, China) and penicillin‒streptomycin (Sigma‒Aldrich, St. Louis, MO). H22, NCI-H82, and NCI-H1975 cells were cultured in RPMI 1640 medium (Gibco, Waltham, USA) supplemented with 10% FBS and 1% penicillin–streptomycin. All the cells were cultured in a humidified atmosphere of 5% CO_2_ at 37 °C. Azvudine (MedChemExpress, New Jersey, USA, 10 µM for 24 h) or Paxlovid (MedChemExpress, New Jersey, USA, 10 µM for 24 hours; nirmatrelvir & ritonavir at a mass ratio of 3:1) was added to the medium when the cells reached the proper density for further experimentation.

### Cell viability assay

Cell viability was assessed using the CellTiter-Glo^®^ luminescent cell viability assay (CTG). Briefly, ~1000 cells were seeded on 96-well plates and treated with azvudine or Paxlovid for 5 days. Then, CellTiter-Glo reagent (Promega, USA) was added to each well, and the cells were incubated for 10 min at room temperature. Luminescence was then measured with a luminometer.

### Colony formation assay

Hep3b or Huh7 cells were seeded into six-well plates at a density of 1000 cells per well and then treated with azvudine (10 μM) or Paxlovid (10 μM). After 72 h of incubation, the medium was replaced with complete DMEM, and the cells were cultured for an additional 14 days at 37 °C. The colonies were then fixed with 4% paraformaldehyde for 15 min and stained with 0.1% crystal violet for 30 min. Finally, the colonies were photographed and counted.

### Invasion assays

Matrigel was diluted with serum-free cell culture medium at a ratio of 1:8 (60 µl per well) and placed on the upper chambers of transwell inserts. Overnight-starved Hep3b or Huh7 cells were resuspended in a serum-free medium, and 1 × 10^5^ cells were added to the upper chambers. Subsequently, 600 µl of medium supplemented with 10% FBS was added to the lower chambers. After 6 h, azvudine or Paxlovid was added to the culture medium. After 48 h of culture, the Transwell inserts were removed, and the invading cells were fixed in 4% paraformaldehyde for 15 min and then stained with 0.1% crystal violet for 30 min. Finally, the cells in the upper chamber were removed, and the number of cells that passed through the Matrigel and the membrane were counted using a light microscope (Olympus, Japan).

### Animal experiments

Four- to six-week-old male BALB/c mice weighing 20.0 ± 2 g were purchased from BEJING HFK BIOSCIENCE Co., Ltd. (Beijing, China). The mice were housed in a specific pathogen-free environment. Each mouse was subcutaneously injected with 5 × 10^5^ H22 cells in the right flank. When the tumors reached ~100 mm^3^, the mice were randomly divided into three groups to receive oral treatment: the azvudine group (1 mg/kg, QD), the Paxlovid group (61.65 mg/kg nirmatrelvir and 20.55 mg/kg ritonavir, BID), and the control group (solvent). The solvent was prepared by sequentially adding the following reagents: 5% DMSO, 40% PEG300, 5% Tween-80, and 50% saline. Tumor volumes were measured daily using a caliper and calculated with the following formula: volume = (length × width^2^)/2. The mice were euthanized when the tumor volume exceeded 1500 mm^3^ or if the maximum tumor diameter exceeded 15 mm.

### Immunohistochemistry

The details of the IHC procedure have been described previously.^49^ The primary antibodies used were as follows: rabbit polyclonal antibody against Ki-67 (ab15580, Abcam), mouse monoclonal PC10 antibody against PCNA (ab29, Abcam).

### Single-cell RNA sequencing

Following the manufacturer’s instructions, we prepared a single-cell suspension via a tumor dissociation kit (Miltenyi Biotec, Germany). After quality inspection, a single-cell suspension with a cell viability of more than 80%, a clumping rate of less than 10%, background debris of less than 50%, and a moderate cell concentration was obtained. Subsequently, the cell barcode-labeled beads and cells were passed through the 10X Genomics platform, and the cells and enzymes were encapsulated with oil droplets to form gel beads in the emulsion. The cells were lysed within each independent oil droplet, and the poly-A of the intracellular mRNA was labeled with a cell barcode on the bead. Reverse transcription was performed to generate cDNA. Finally, the oil phase was removed, and the next-generation sequencing library was constructed. Finally, sequencing was performed using the NovaSeq 6000 platform (Illumina, United States). Cell Ranger 8.0.1 software was used to map reads to a reference genome and quantify gene expression levels. Data analysis and clustering were performed using the “Seurat” R package of R software (version 4.3.3). Cell-to-cell communication analysis was performed using the R software package “CellChat”.

### Statistical analysis

The statistical analysis was calculated by R version 4.0.3. Statistical significance was defined as a two-sided *p* value of <0.05. Continuous variables are presented as the means (standard deviations) for normally distributed data or medians (interquartile ranges) for nonnormally distributed data. Differences between groups in continuous variables were assessed using the independent *t*-test or Mann–Whitney *U*- test, as appropriate. Categorical variables are presented as counts and percentages, with differences between groups evaluated using the chi-square test. Multiple imputation was employed to handle missing values.

To diminish the influence of confounding variables on the evaluation of interventions, we used greedy matching with a 1:2 ratio to control for baseline covariates (age, sex, severity, BMI, COVID-19 vaccination dose, concomitant antibiotics, concomitant systemic steroids, time from diagnosis to treatment exposure, comorbidities, laboratory results) between azvudine and Paxlovid groups using the logistic regression model. The balance of baseline covariates between the groups was assessed using p values and SMDs, with *p* > 0.05 and SMD <0.1 indicating an optimal balance between the groups.

Cumulative event curves were constructed by the Kaplan‒Meier analysis and the survival difference between groups was determined by the log-rank test. Cox proportional hazards regression models were constructed to evaluate HRs with 95% CIs for the primary and secondary outcomes, adjusting for all baseline covariates. Subgroup analyses were performed, stratified by sex, age, severity, COVID-19 vaccination dose, concomitant antibiotics or systemic steroid, time from diagnosis to treatment exposure, and comorbidities.

To test the robustness of our findings, we conducted three sensitivity analysis. First, we addressed missing values through mean imputation and then used a logistic regression model to perform a 1:2 greedy match to assess the reliability of the results. Second, we applied a Probit model to perform another 1:2 greedy match. Third, because it takes time for the drug to exert its effects, we excluded patients who were discharged or died on the first day of drug administration based on the time required to reach peak blood concentration to narrow the study population.

## Supplementary information

Supplementary Material

## Data Availability

We deposited the scRNA-seq data in the Gene Expression Omnibus repository (GSE285359). The clinical dataset is available from the corresponding author on reasonable request.
