# Supplementary material for: Real‐world effectiveness and safety of oral azvudine versus nirmatrelvir‒ritonavir (Paxlovid) in hospitalized patients with COVID-19: a multicenter, retrospective, cohort study
Source: Signal Transduct Target Ther. 2025 Jan 17;10:30. doi: 10.1038/s41392-025-02126-w (PMC11739574; doi:10.1038/s41392-025-02126-w)
Supplement: Supplementary file 1 — Supplementary Material [file 41392_2025_2126_MOESM1_ESM.docx]

Supplementary Materials for

Real‐world effectiveness and safety of oral azvudine versus nirmatrelvir-ritonavir (Paxlovid) in hospitalized patients with COVID-19: A multicenter, retrospective, cohort study

Haiyu Wang ^1†^, Guangying Cui ^1†^, Ming Cheng ^2†^, Tuerganaili Aji ^3†^, Guotao Li ^4†^, Xinjun Hu ^5^, Guangming Li ^6^, Shixi Zhang ^7^, Yanyang Zhang ^8^, Linqi Diao ^8^, Pan Li ^1^, Ling Wang ^9^, Yiqiang Yuan ^9^, Guowu Qian ^10^, Ruiqing Zhang ^3^, Xiaoli Jin ^4^, Juan Wang ^6^, Hong Luo ^11^, Donghua Zhang ^12^, Mingming Wang ^7^, Silin Li ^13^, Zhan Song ^10^, Mengzhao Yang ^1^, Guanyue Su ^1^, Ranran Sun ^1^, Junbiao Chang ^14^*, Zujiang Yu^1^*, Zhigang Ren ^1^*

Correspondence to: [changjunbiao@zzu.edu.cn](mailto:changjunbiao@zzu.edu.cn); [johnyuem@zzu.edu.cn](mailto:johnyuem@zzu.edu.cn); [fccrenzg@zzu.edu.cn](mailto:fccrenzg@zzu.edu.cn)

**This PDF file includes:**

Materials and Methods

Figures. S1 to S15

Tables S1 to S5

Study Protocol

**Materials and methods**

**Study population**

Ten hospitals in Henan Province included the First Affiliated Hospital of Zhengzhou University, Henan Provincial Chest Hospital, Henan Infectious Disease Hospital, Luoyang Central Hospital, the First Affiliated Hospital of Henan University of Science & Technology, Nanyang Central Hospital, the Fifth People’s Hospital of Anyang, Shangqiu Municipal Hospital, Guangshan County People’s Hospital, and Fengqiu County People’s Hospital). One hospital in Xinjiang Province was the First Affiliated Hospital of Xinjiang Medical University. The study is registered with ClinicalTrials.gov (NCT06349655).

**Definition of covariates**

The laboratory results collected at diagnosis included neutrophil (Neut), lymphocyte (Lymph), glucose (Glu), high-density lipoprotein (HDL), low-density lipoprotein (LDL), alanine aminotransferase (ALT), aspartate aminotransferase (AST), creatinine (CREA), glomerular filtration rate (GFR), C–reactive protein (CRP), procalcitonin (PCT), prothrombin time (PT), activated partial thromboplastin time (APTT), cholesterol (CH), triglyceride (TG), alkaline phosphatase (ALP), gamma-glutamyl transpeptidase (GGT), albumin (ALB), and total bilirubin (TBIL) levels. Comorbidities, including diabetes, hypertension, liver diseases, cardio-cerebral diseases, kidney diseases, primary malignant tumors, chronic respiratory diseases, and autoimmune diseases, were obtained from electronic records.

**Statistical analysis**

The proportional hazards assumption was assessed using Schoenfeld residuals. If the variables did not satisfy this assumption, time-dependent covariables were constructed to control for the effect of time. Multicollinearity was tested using the VIF, with a VIF value > 5 indicating the presence of multicollinearity.

**Supplementary Figures S1 to S15**


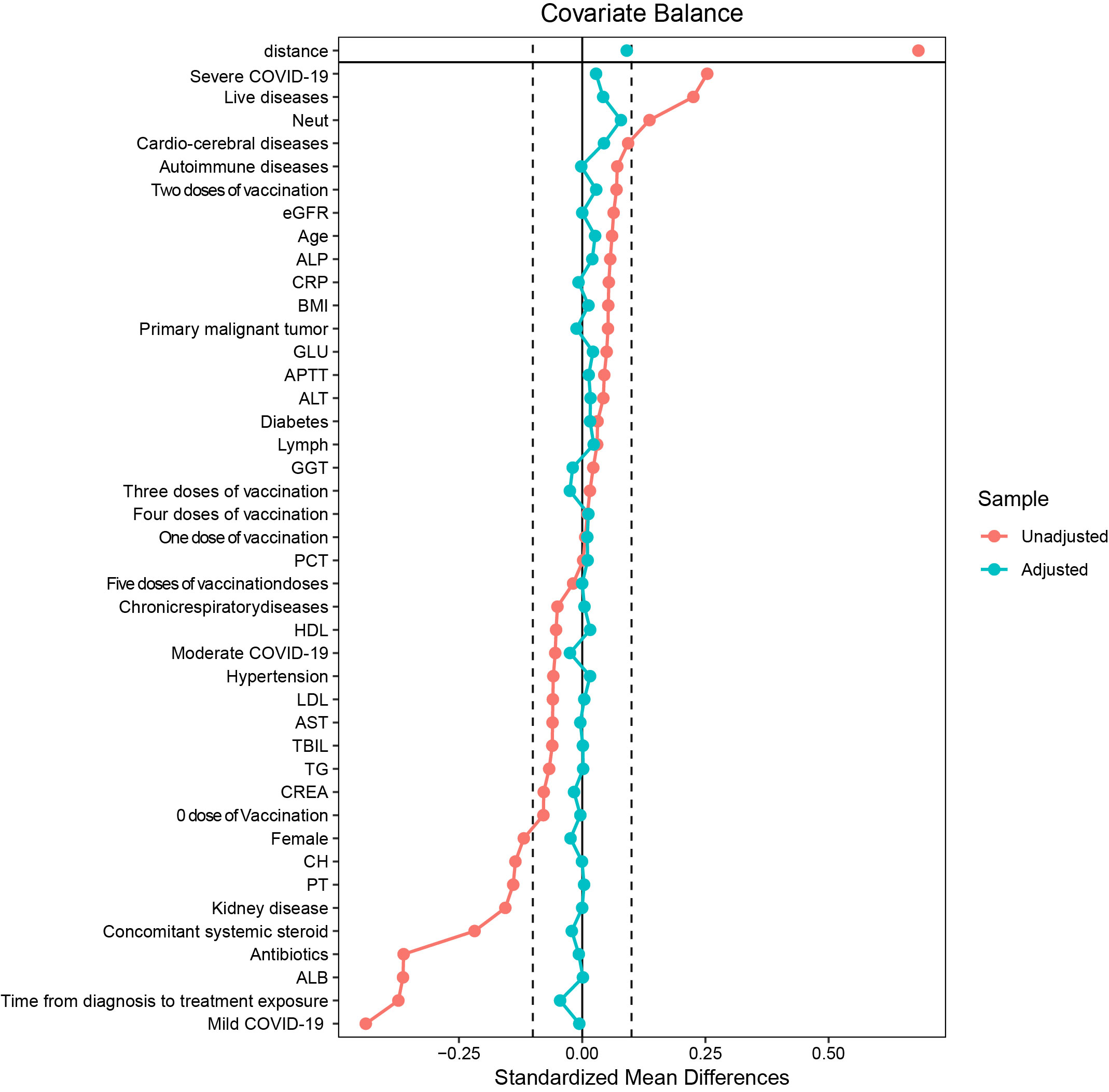
**Figure. S1. Baseline characteristics of patients in Henan cohort before and after propensity score matching.**


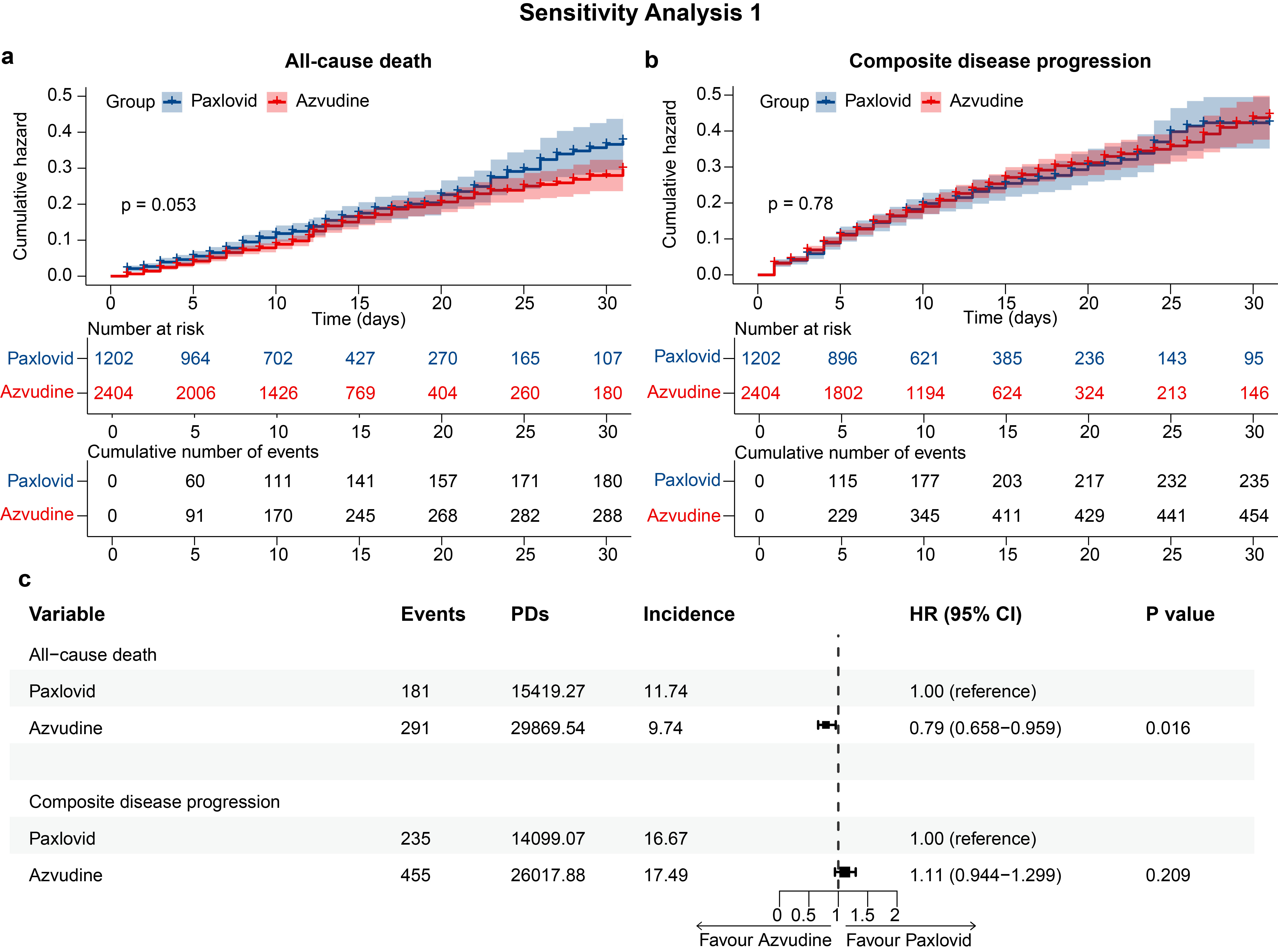


**Figure. S2. Kaplan–Meier curves and multivariate Cox proportional hazards regression analysis of patients receiving azvudine versus Paxlovid from Henan cohort in the sensitivity analysis where the missing data was filled up with the mean value.** Cumulative hazard of all‐cause death (a) and composite disease progression (b) assessed by Kaplan–Meier curves. (c) Hazard ratio of all-cause death and composite disease progression after adjusting for all baseline covariates in Table 1. HR: Hazard Ratio; 95%CI: 95% confidence interval. PDs: Person-days. Incidence: events/per 1000 PDs.


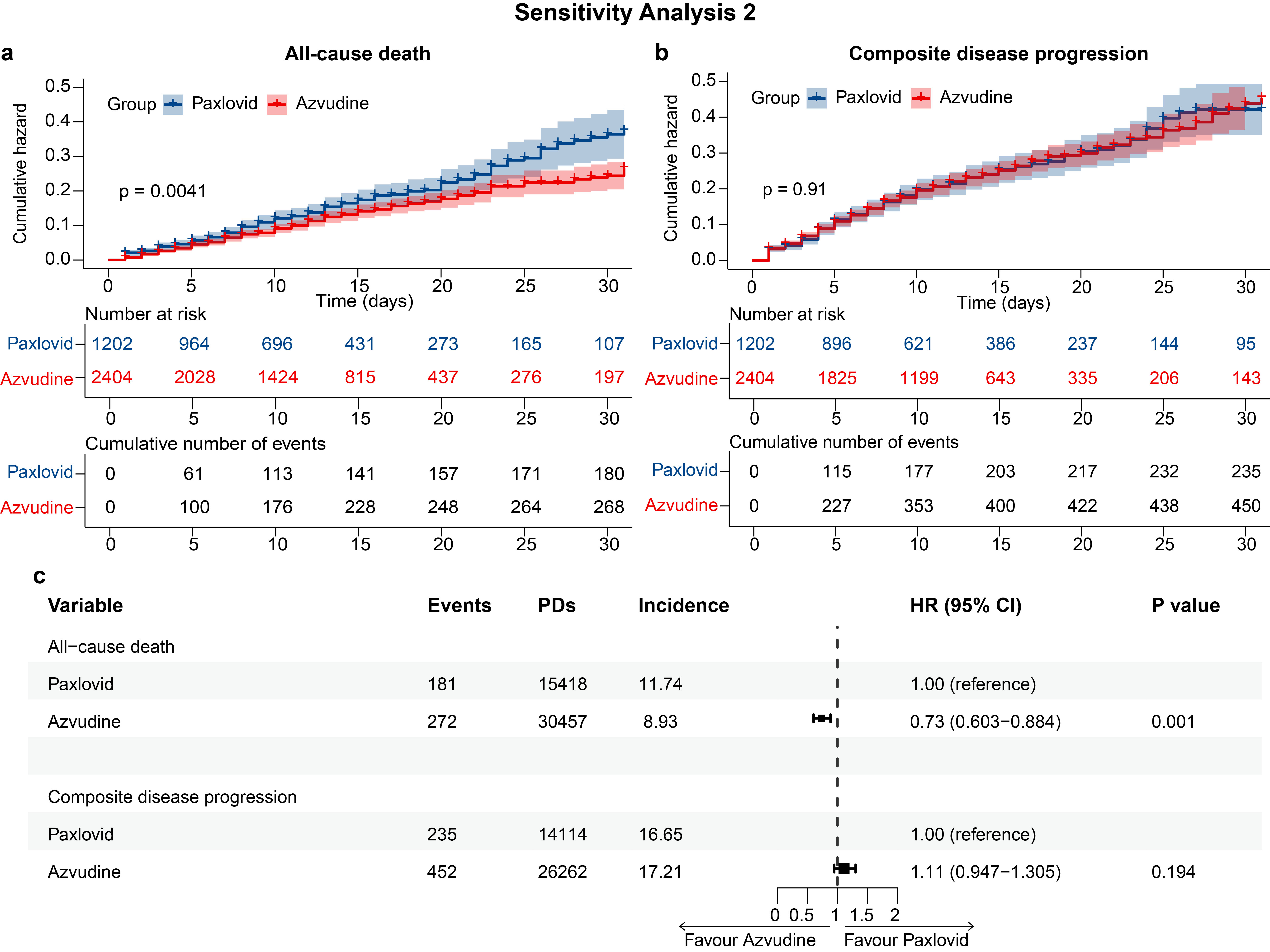


**Figure. S3. Kaplan–Meier curves and Cox proportional hazards regression analysis of patients receiving azvudine versus Paxlovid from Henan cohort in the sensitivity analysis where propensity score matching was performed using Probit model.** Cumulative hazard of all‐cause death (a) and composite disease progression (b) assessed by Kaplan–Meier curves. (c) Hazard Ratio of all-cause death and composite disease progression after adjusting for all baseline covariates in Table 1. HR: Hazard Ratio; 95%CI: 95% confidence interval. PDs: Person-days. Incidence: events/per 1000 PDs.


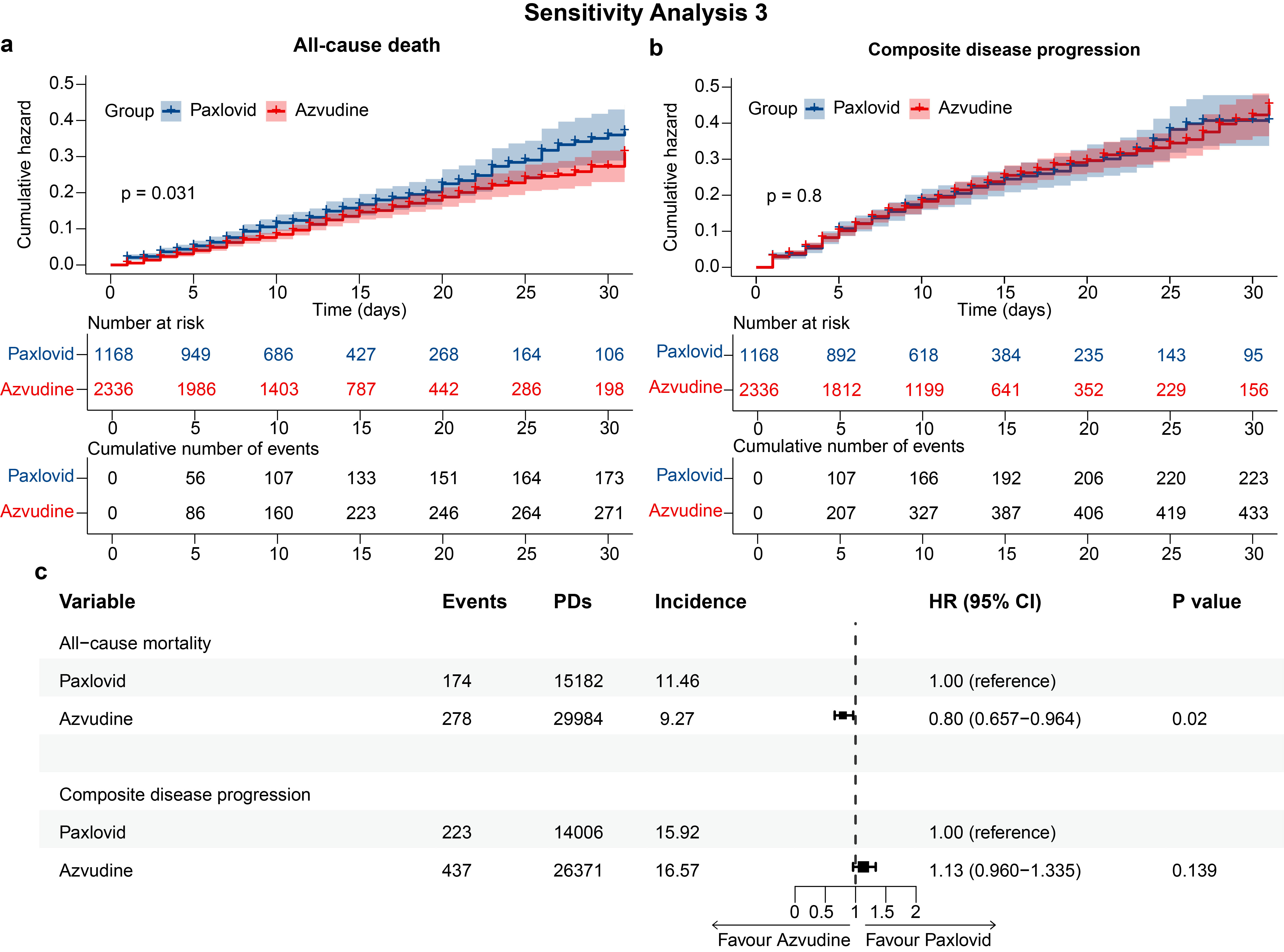


**Figure. S4.** **Kaplan–Meier curves and Cox proportional hazards regression analysis of patients receiving azvudine versus Paxlovid** **from Henan cohort in the sensitivity analysis where patients who discharged within one day after receiving antiviral treatment were excluded.** Cumulative hazard of all‐cause death (a) and composite disease progression (b) assessed by Kaplan–Meier curves. (c) Hazard Ratio of all-cause death and composite disease progression after adjusting for all baseline covariates in Table 1. HR: Hazard Ratio; 95%CI: 95% confidence interval. PDs: Person-days. Incidence: events/per 1000 PDs.

**
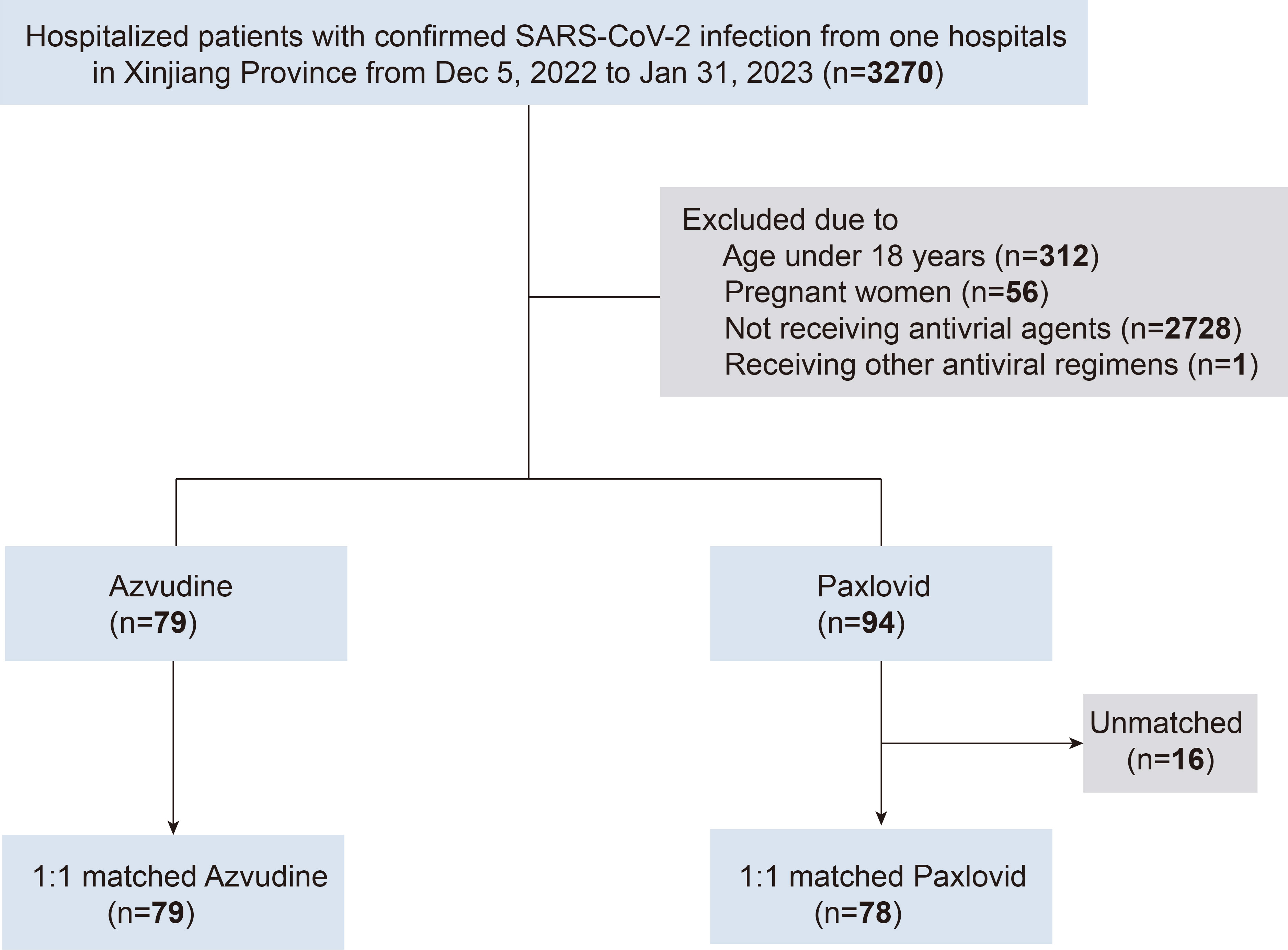
**

**Figure. S5.** **The flowchart of Xinjiang cohort study design.**

**
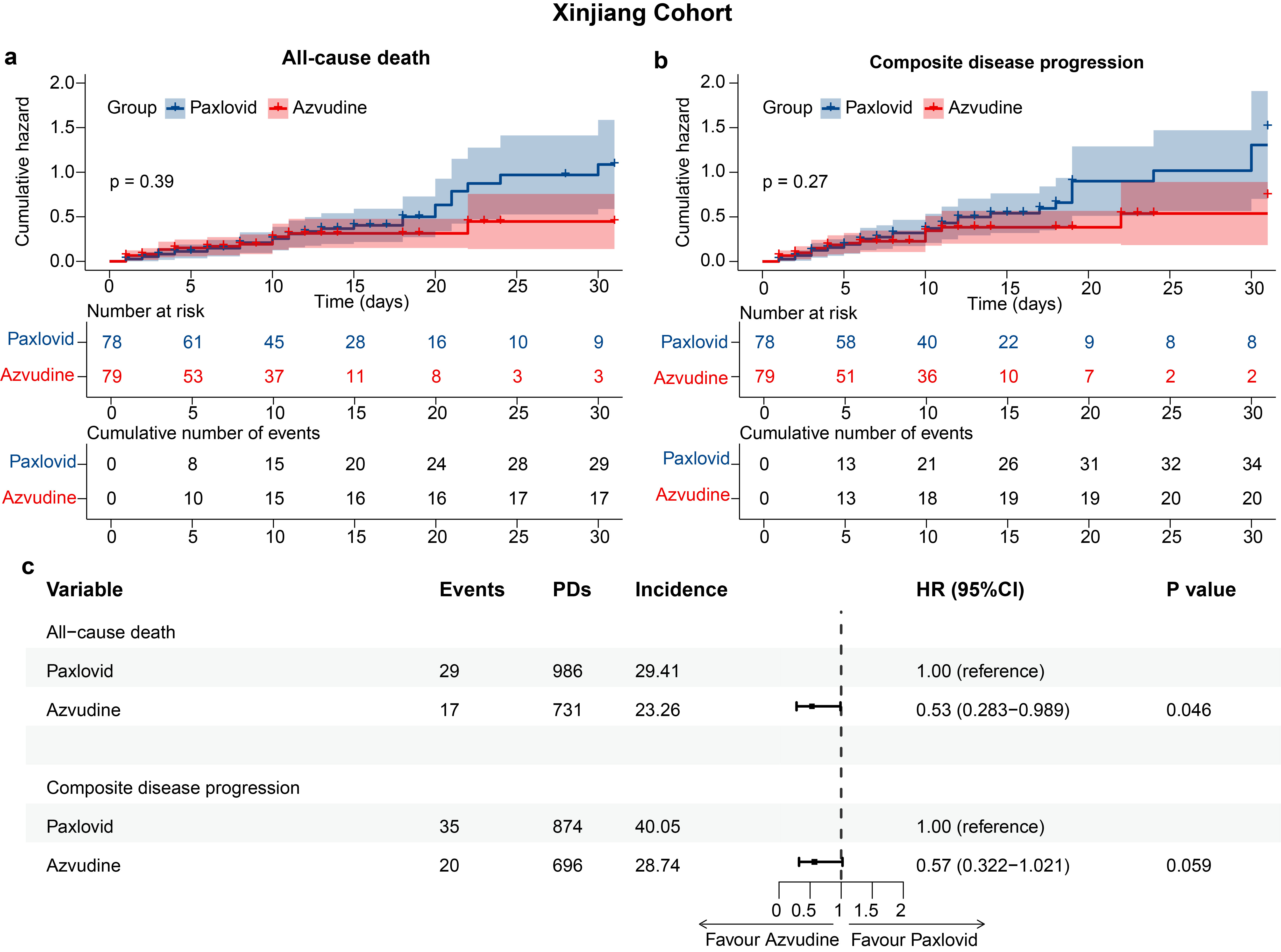
**

**Figure. S6.** **Kaplan–Meier curves and multivariate Cox proportional hazards regression analysis of patients receiving azvudine treatment versus Paxlovid treatment in Xinjiang cohort.** Cumulative hazard of all‐cause death (a) and composite disease progression (b) assessed by Kaplan–Meier curves. (c) Hazard ratio of all-cause death and composite disease progression after adjusting for all baseline covariates in Table S5. HR: Hazard ratio; 95% CI: 95% confidence interval. PDs: Person-days. Incidence: events/per 1000 PDs.

**
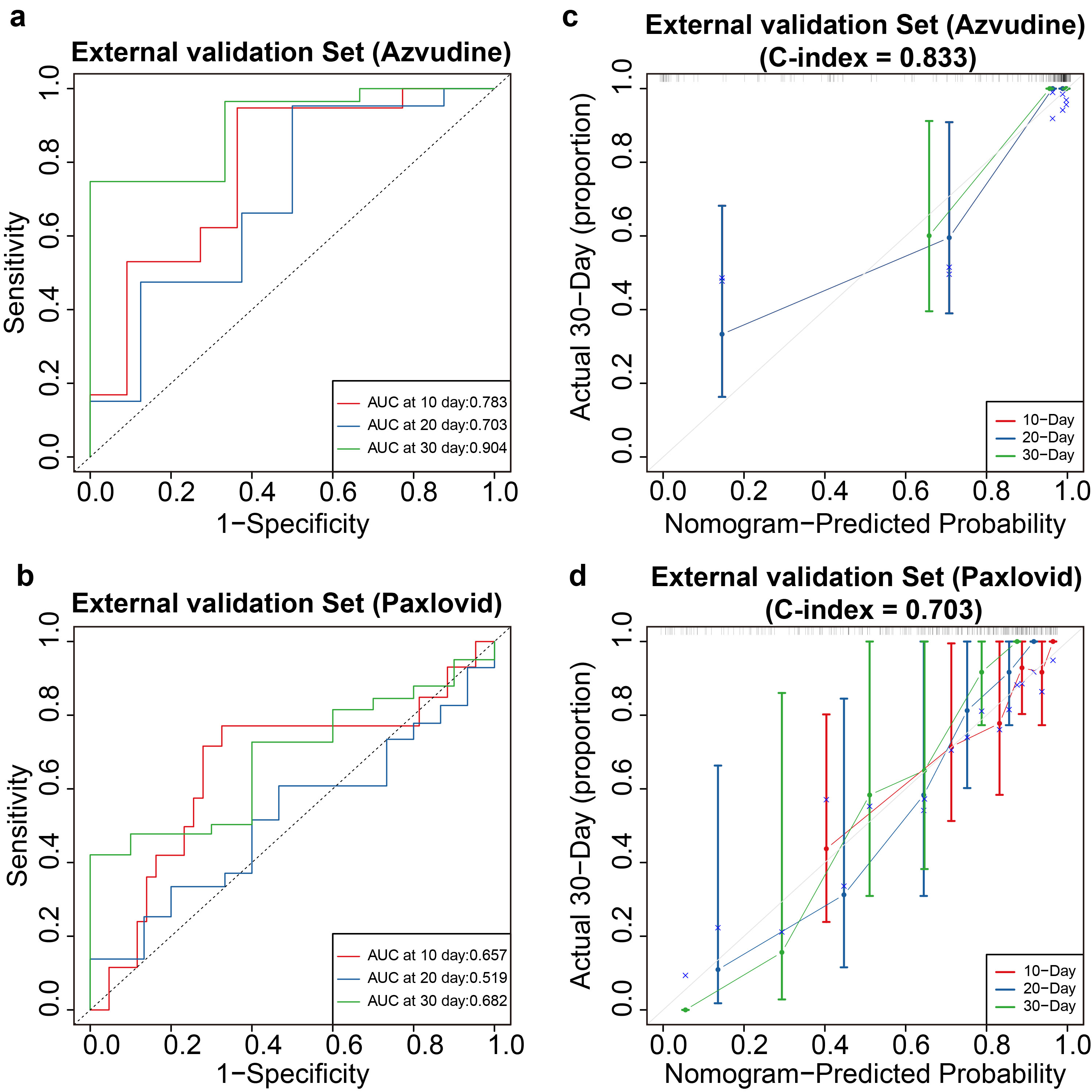
**

**Figure. S7. Receiver operating characteristic curve (ROC) and calibration curves in external validation set (Xinjiang cohort).** ROC curves of nomogram for the survival prediction of patients with azvudine treatment (a) or Paxlovid treatment (b) in external validation set. Calibration curves of nomogram for the survival prediction of patients with azvudine treatment (c) or Paxlovid treatment (d) in external validation set.


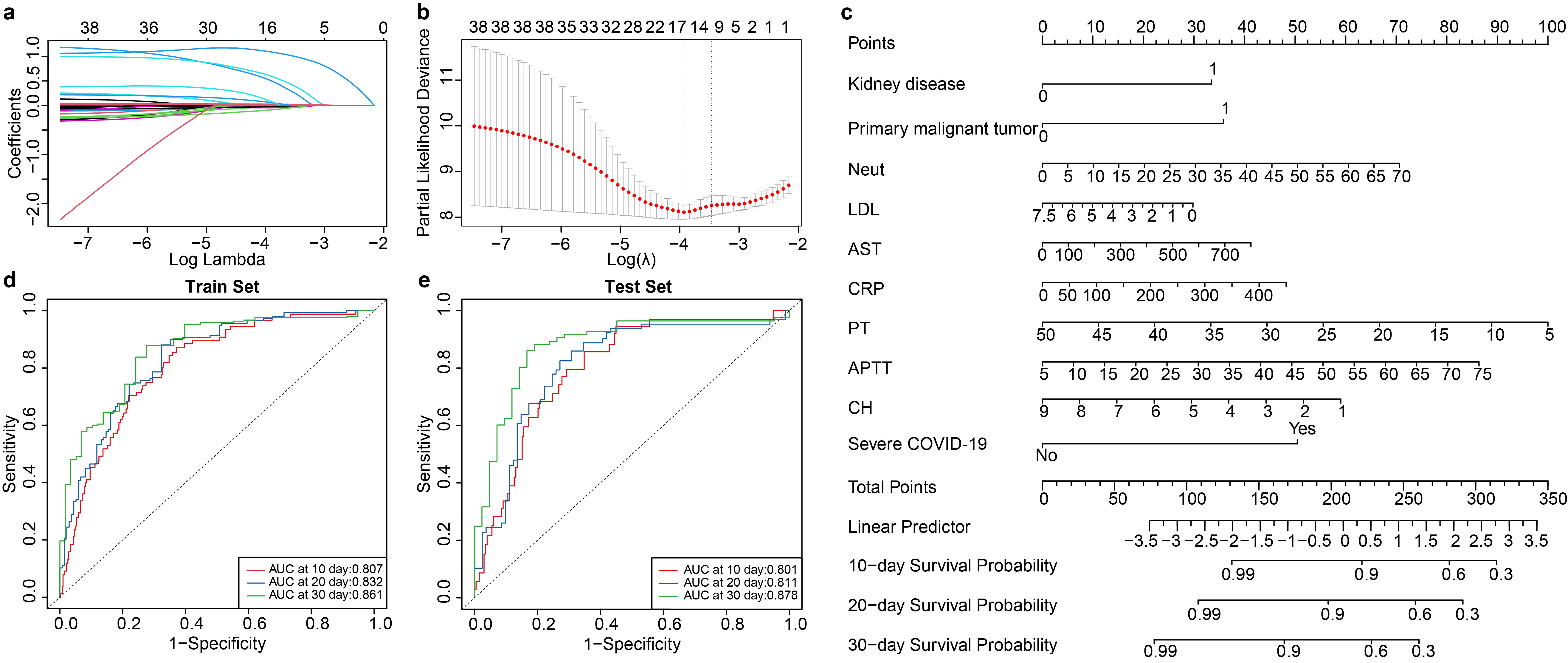


**Figure. S8. A nomogram for survival prediction of hospitalized COVID-19 patients receiving Paxlovid in Henan cohort.** (a) LASSO coefficient profile of 40 features. (b) Tuning parameter (λ) selection in the LASSO model used fivefold cross-validation. (c) Nomogram to estimate 10-, 20-, and 30-day survival for COVID-19patients receiving azvudine. The prediction performance of LASSO model evaluated by ROC curves in the training set (d) and test set (e).


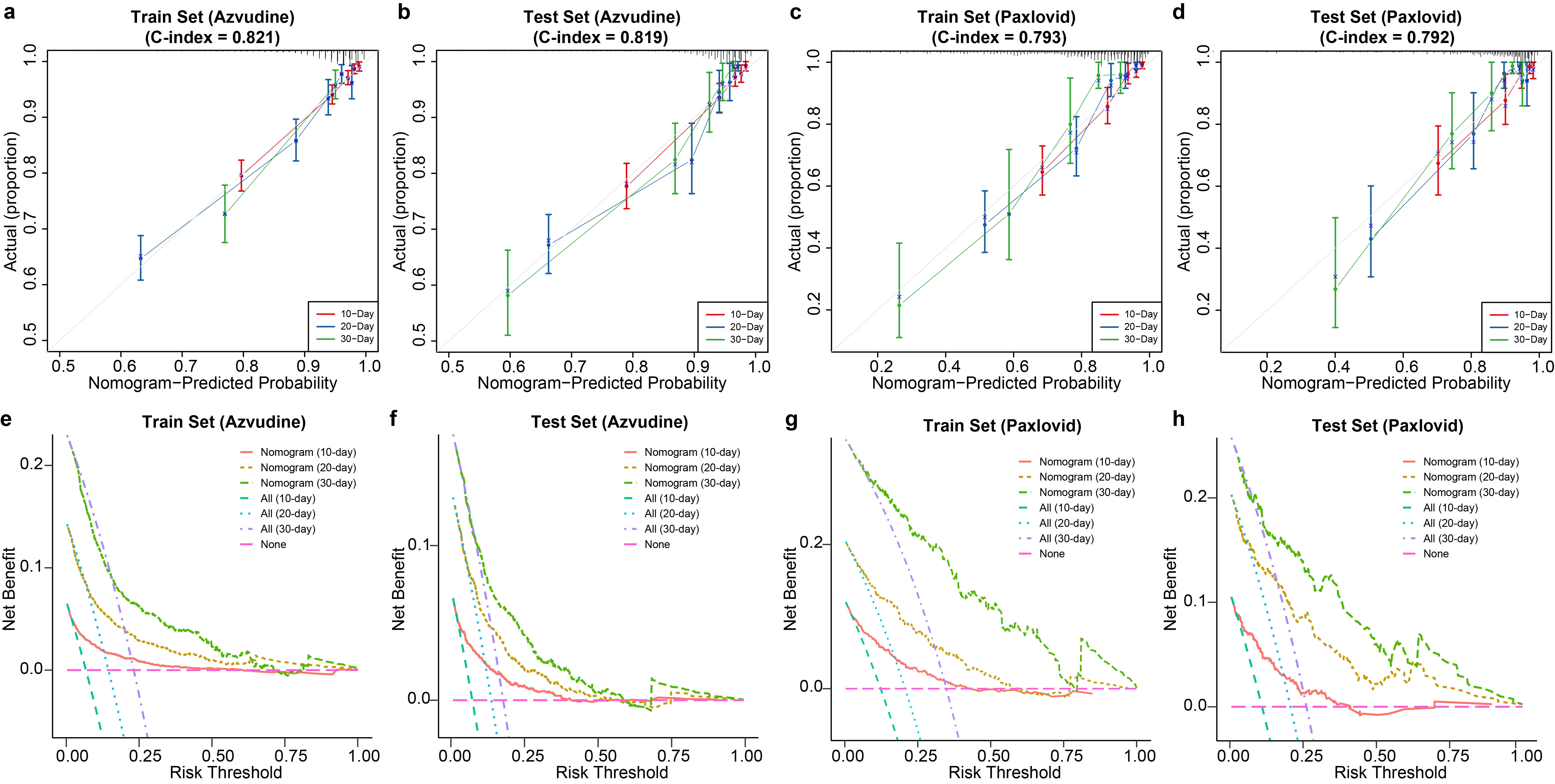


**Figure. S9.** **Calibration curves and decision curve analysis in Henan cohort.** Calibration curves of nomogram for the survival prediction of patients with azvudine treatment (a-b) or Paxlovid treatment (c-d) in both training and test set. Decision curve analysis of nomogram for the survival prediction of patients with azvudine (e-f) or Paxlovid (g-h) in both training and test set.

**
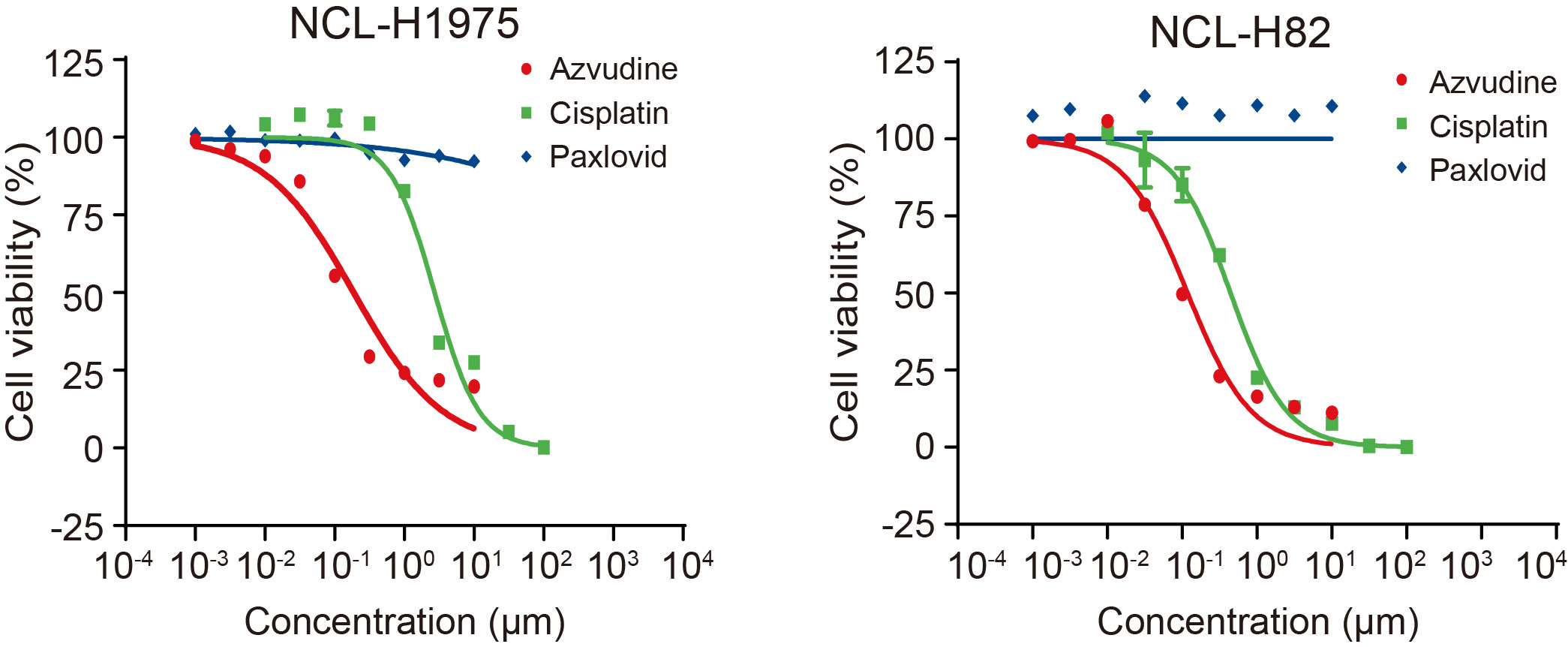
**

**Figure. S10.** The proliferation ability of NCI-H1975 and NCI-H82 cells with azvudine, Paxlovid, and Cisplatin were assessed by CTG assay.


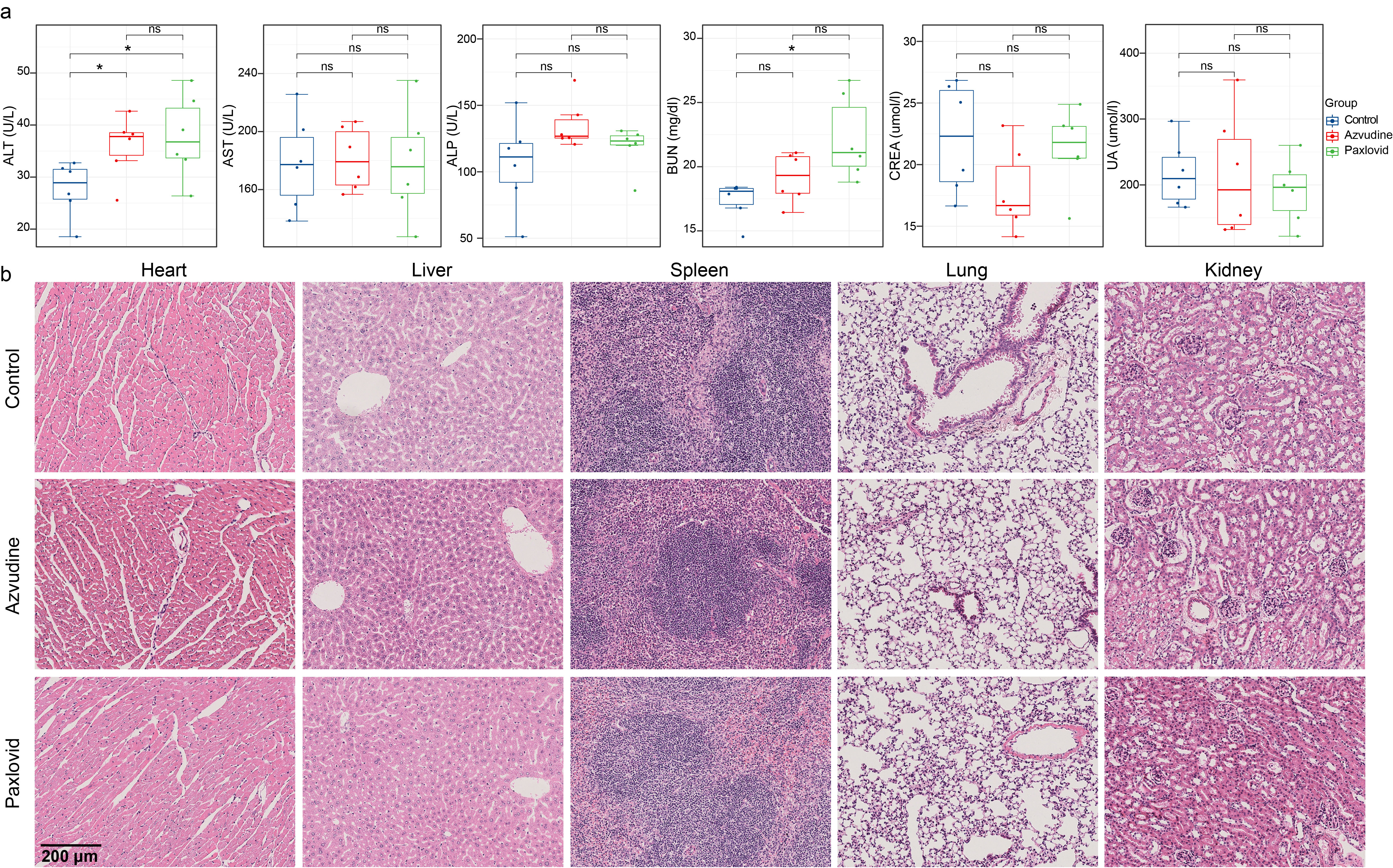


**Figure. S11.** **Safety analysis.** (a) Liver and kidney function in mice. (b) H&E staining images of the heart, liver, spleen, lungs, and kidneys in tumor-bearing mice. Scar bar, 200μm.

**
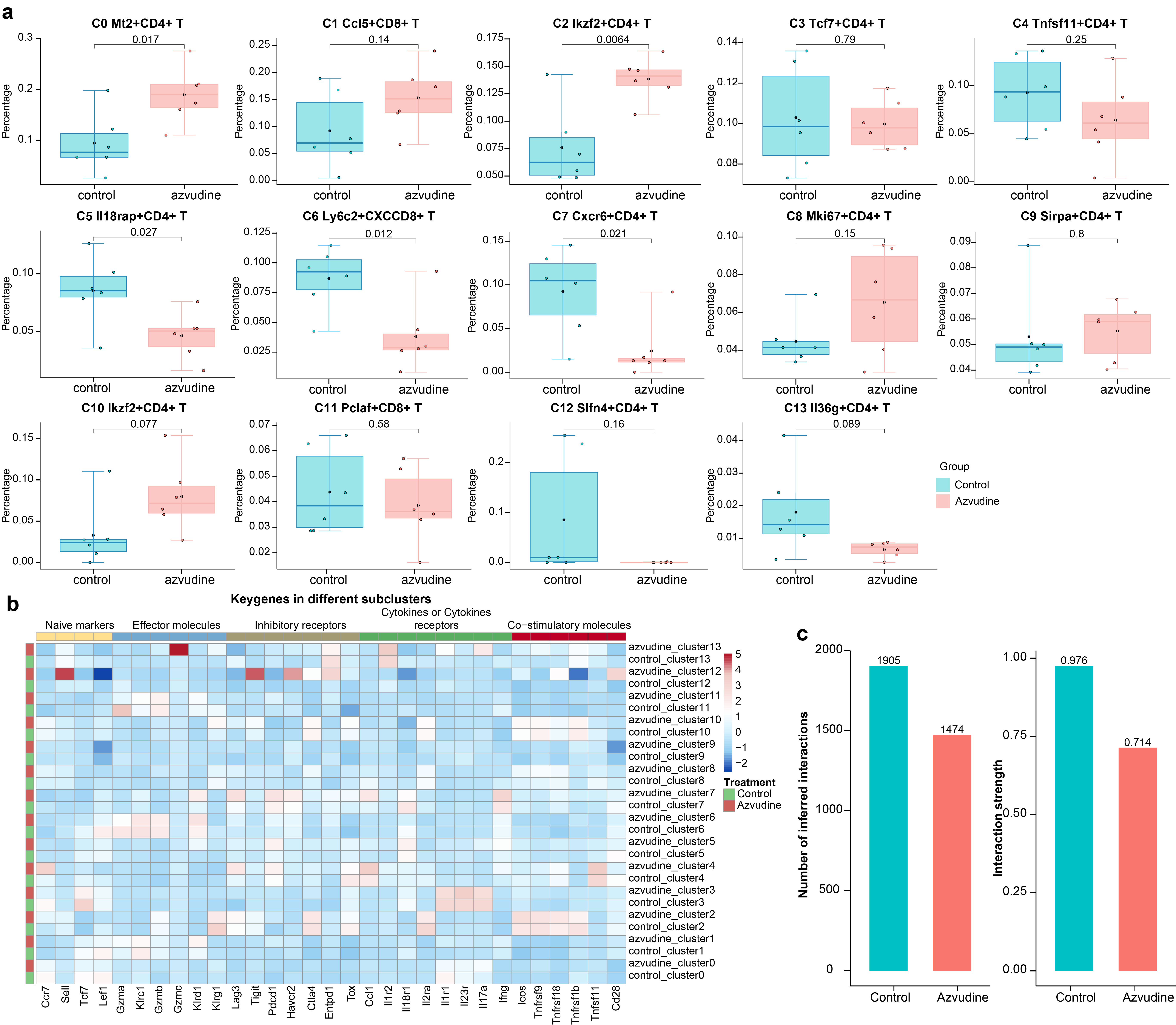
**

**Figure. S12. scRNA-seq confirmed the phenotypical heterogeneity of T lymphocytes in the tumor immune microenvironment of HCC.** (a) Differences in the proportion of distinct T lymphocyte clusters in the control and azvudine groups. (b) The expression levels of characteristic genes across 14 clusters of T lymphocytes in the control and azvudine groups. (c) Bar plot presents the interaction number and strength of the control and azvudine groups. C: cluster.


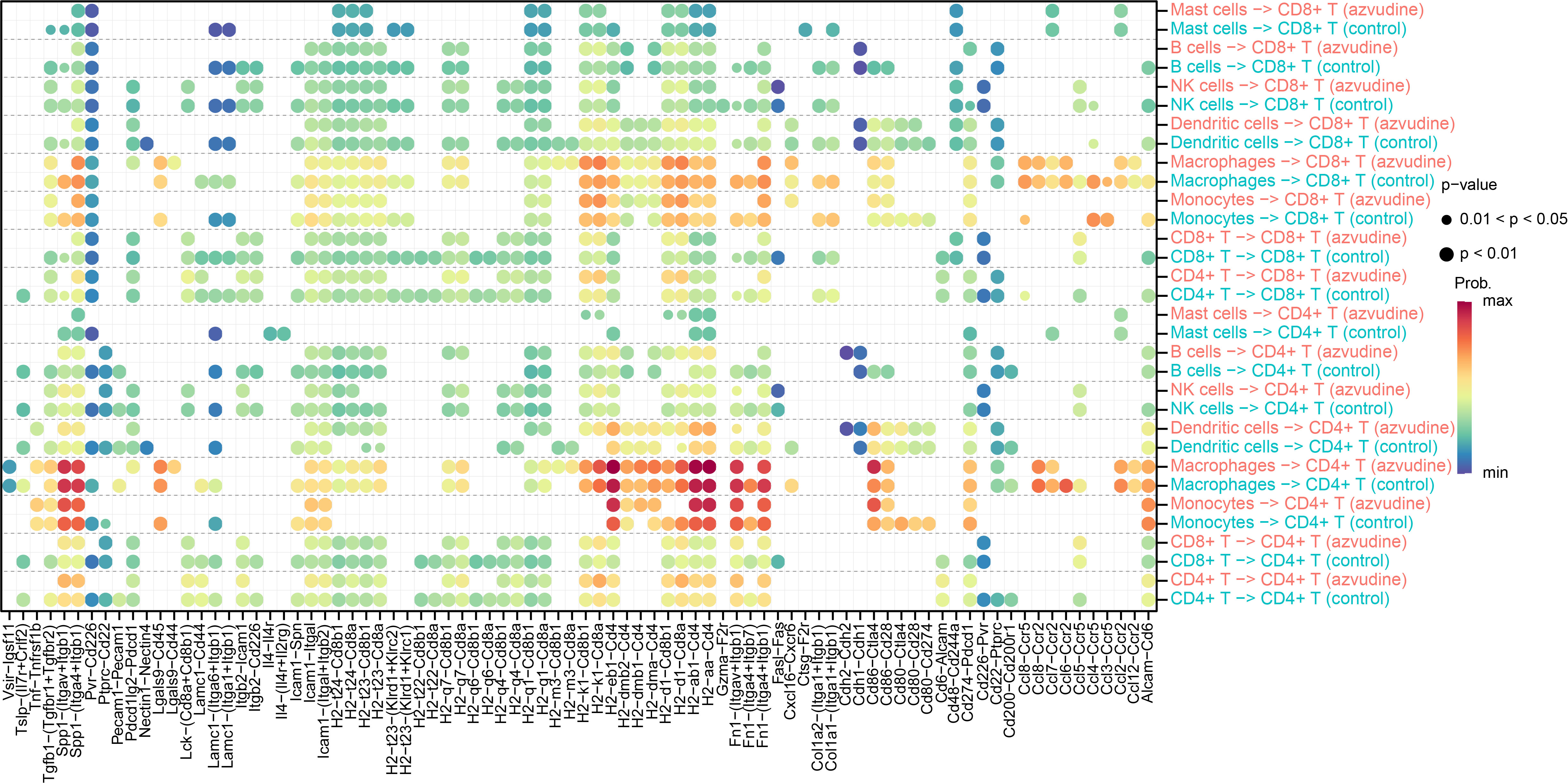


**Figure. S13. Bubble map presenting the comparison of significant ligand–receptor pairs in the control and azvudine groups, when CD4^+^ T and CD8^+^ T cells as receptor cells**.


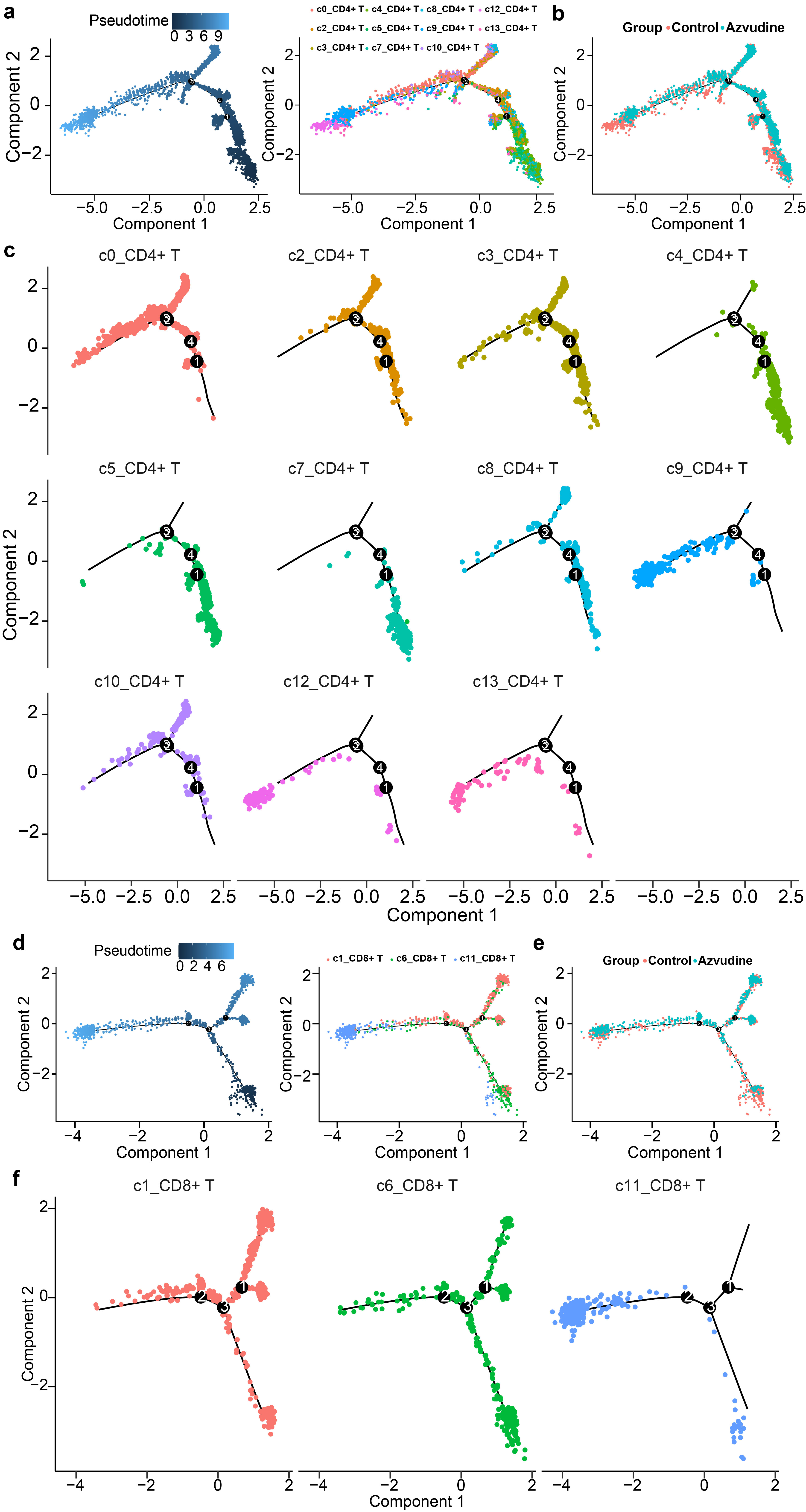


**Figure. S14. Pseudotime analysis and development trajectories of T lymphocyte clusters.** (a) Pseudotime trajectory of CD4^+^ T cells. (b) Cell trajectories of CD4^+^ T cells in the azvudine and control groups. (c) Mapping of each CD4^+^ T cell subcluster on a trajectory diagram. (d) Pseudotime trajectory of CD8^+^ T cells. (e) Cell trajectories of CD8^+^ T cells in the azvudine and control groups. (f) Mapping of each CD8^+^ T cell subcluster on a trajectory diagram.


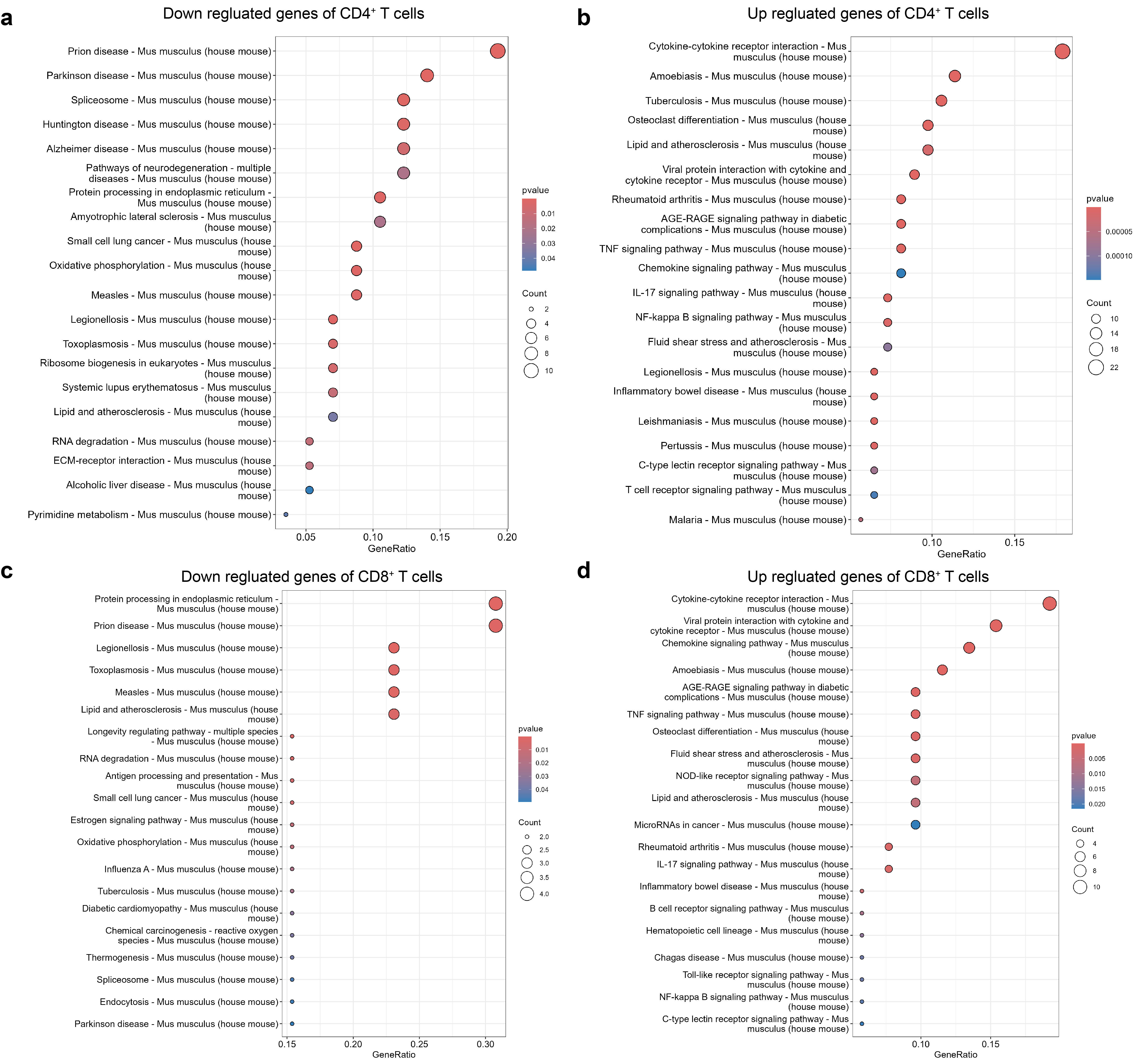


**Figure. S15. KEGG pathway enrichment analysis in CD4^+^ and CD8^+^ T cells**. The top 20 KEGG pathways of differentially down-regulated genes (a) and up- regulated genes (b) in CD4^+^ T cells. The top 20 KEGG pathways of differentially down-regulated genes (c) and up- regulated genes (d) in CD8^+^ T cells.

**Supplementary Tables S1 to S5**

**Table S1. Baseline characteristics of patients with SARS-CoV-2 infection before and after propensity score matching from Henan cohort in the sensitivity analysis where the missing data was filled up with the mean value.**

| **Characteristics** | **Before matching** | | |  | **After 2:1 matching** | | |
| --- | --- | --- | --- | --- | --- | --- | --- |
|  | **Azvudine (n=6943)** | **Paxlovid (n=1202)** | **P value** |  | **Azvudine (n=2404)** | **Paxlovid (n=1202)** | **P value** |
| **Age, mean (SD), year** | 68.37 (15.21) | 69.26 (14.81) | 0.059 |  | 68.97 (14.37) | 69.26 (14.81) | 0.56 |
| **Gender, n (%)** |  |  | <0.001 |  |  |  | 0.224 |
| **Male** | 4170 (60.1) | 790 (65.7) |  |  | 1529 (63.6) | 790 (65.7) |  |
| **Female** | 2773 (39.9) | 412 (34.3) |  |  | 875 (36.4) | 412 (34.3) |  |
| **BMI, mean (SD), kg/m^2^** | 24.22 (1.94) | 24.37 (2.66) | 0.025 |  | 24.33 (2.35) | 24.37 (2.66) | 0.654 |
| **Severity at admission, n (%)** |  |  | <0.001 |  |  |  | 0.651 |
| **Mild** | 977 (14.1) | 57 (4.7) |  |  | 110 (4.6) | 57 (4.7) |  |
| **Moderate** | 4459 (64.2) | 740 (61.6) |  |  | 1518 (63.1) | 740 (61.6) |  |
| **Severe** | 1507 (21.7) | 405 (33.7) |  |  | 776 (32.3) | 405 (33.7) |  |
| **Vaccination doses, n (%)** |  |  | 0.072 |  |  |  | 0.809 |
| **0 dose** | 2229 (32.1) | 342 (28.5) |  |  | 704 (29.3) | 342 (28.5) |  |
| **1 dose** | 416 (6.0) | 75 (6.2) |  |  | 139 (5.8) | 75 (6.2) |  |
| **2 doses** | 923 (13.3) | 191 (15.9) |  |  | 350 (14.6) | 191 (15.9) |  |
| **3 doses** | 3312 (47.7) | 582 (48.4) |  |  | 1185 (49.3) | 582 (48.4) |  |
| **4 doses** | 61 (0.9) | 12 (1.0) |  |  | 26 (1.1) | 12 (1.0) |  |
| **5 doses** | 2 (0.0) | 0 (0.0) |  |  | 0 (0.0) | 0 (0.0) |  |
| **Time from diagnosis to treatment exposure, n (%)** |  |  | <0.001 |  |  |  | 0.243 |
| **> 5 days** | 1136 (16.4) | 409 (34.0) |  |  | 770 (32.0) | 409 (34.0) |  |
| **0–5 days** | 5807 (83.6) | 793 (66.0) |  |  | 1634 (68.0) | 793 (66.0) |  |
| **Concomitant antibiotics, n (%)** |  |  | <0.001 |  |  |  | 0.932 |
| **No** | 3070 (44.2) | 743 (61.8) |  |  | 1481 (61.6) | 743 (61.8) |  |
| **Yes** | 3873 (55.8) | 459 (38.2) |  |  | 923 (38.4) | 459 (38.2) |  |
| **Concomitant** **systemic steroid, n (%)** |  |  | <0.001 |  |  |  | 0.99 |
| **No** | 3875 (55.8) | 795 (66.1) |  |  | 1588 (66.1) | 795 (66.1) |  |
| **Yes** | 3068 (44.2) | 407 (33.9) |  |  | 816 (33.9) | 407 (33.9) |  |
| **Comorbidities, n (%)** |  |  |  |  |  |  |  |
| **Diabetes** | 1764 (25.4) | 322 (26.8) | 0.328 |  | 616 (25.6) | 322 (26.8) | 0.477 |
| **Hypertension** | 3106 (44.7) | 503 (41.8) | 0.067 |  | 976 (40.6) | 503 (41.8) | 0.495 |
| **Liver diseases** | 770 (11.1) | 242 (20.1) | <0.001 |  | 444 (18.5) | 242 (20.1) | 0.248 |
| **Cardio-cerebral diseases** | 2196 (31.6) | 434 (36.1) | 0.002 |  | 833 (34.7) | 434 (36.1) | 0.409 |
| **Kidney diseases** | 1864 (26.8) | 247 (20.5) | <0.001 |  | 477 (19.8) | 247 (20.5) | 0.649 |
| **Primary malignant tumor** | 730 (10.5) | 147 (12.2) | 0.085 |  | 285 (11.9) | 147 (12.2) | 0.843 |
| **Chronic respiratory diseases** | 1164 (16.8) | 180 (15.0) | 0.133 |  | 380 (15.8) | 180 (15.0) | 0.548 |
| **Autoimmune diseases** | 234 (3.4) | 59 (4.9) | 0.01 |  | 114 (4.7) | 59 (4.9) | 0.89 |
| **Laboratory parameters, mean (SD)** |  |  |  |  |  |  |  |
| **Neutrophil, ×10^9^/L** | 5.81 (4.60) | 6.40 (4.31) | <0.001 |  | 6.27 (5.66) | 6.40 (4.31) | 0.498 |
| **Lymphocyte, ×10^9^/L** | 1.18 (2.23) | 1.26 (3.50) | 0.271 |  | 1.20 (2.42) | 1.26 (3.50) | 0.550 |
| **Glucose, mmol/L** | 8.02 (3.66) | 8.31 (3.66) | 0.01 |  | 8.14 (3.84) | 8.31 (3.66) | 0.201 |
| **High-density lipoprotein, mmol/L** | 1.17 (1.68) | 1.10 (0.24) | 0.119 |  | 1.16 (1.66) | 1.10 (0.24) | 0.206 |
| **Low-density lipoprotein, mmol/L** | 2.35 (1.73) | 2.28 (0.67) | 0.145 |  | 2.34 (1.72) | 2.28 (0.67) | 0.245 |
| **Alanine aminotransferase, IU/L** | 36.91 (94.98) | 40.12 (62.36) | 0.259 |  | 38.88 (114.39) | 40.12 (62.36) | 0.726 |
| **Aspartate aminotransferase, IU/L** | 42.92 (151.83) | 41.07 (49.03) | 0.676 |  | 40.28 (90.01) | 41.07 (49.03) | 0.777 |
| **Creatine, μmol/L** | 108.42 (171.96) | 97.19 (108.34) | 0.029 |  | 97.16 (123.24) | 97.19 (108.34) | 0.994 |
| **Glomerular filtration rate, ml/min** | 81.46 (23.65) | 83.28 (42.21) | 0.032 |  | 82.41 (36.76) | 83.28 (42.21) | 0.524 |
| **C-reactive protein, mg/L** | 54.65 (47.75) | 58.51 (52.46) | 0.011 |  | 59.44 (54.42) | 58.51 (52.46) | 0.626 |
| **Procalcitonin, ng/ml** | 1.22 (5.63) | 1.25 (4.90) | 0.902 |  | 1.30 (6.44) | 1.25 (4.90) | 0.793 |
| **Prothrombin time, s** | 16.91 (8.81) | 15.45 (7.98) | <0.001 |  | 15.47 (7.09) | 15.45 (7.98) | 0.926 |
| **Activated partial thromboplastin time, s** | 27.52 (10.06) | 27.91 (11.59) | 0.219 |  | 28.20 (8.95) | 27.91 (11.59) | 0.417 |
| **Cholesterol, mmol/L** | 4.02 (1.75) | 3.93 (0.81) | 0.078 |  | 3.98 (1.74) | 3.93 (0.81) | 0.275 |
| **Triglyceride, mmol/L** | 1.49 (1.78) | 1.43 (0.60) | 0.217 |  | 1.49 (1.79) | 1.43 (0.60) | 0.237 |
| **Alkaline phosphatase, IU/L** | 82.30 (49.15) | 87.54 (88.26) | 0.003 |  | 85.32 (55.77) | 87.54 (88.26) | 0.357 |
| **Gamma-glutamyl transpeptidase, IU/L** | 57.60 (96.97) | 61.32 (64.79) | 0.201 |  | 61.54 (130.25) | 61.32 (64.79) | 0.955 |
| **Albumin, g/L** | 36.50 (25.32) | 33.56 (7.30) | <0.001 |  | 33.27 (12.43) | 33.56 (7.30) | 0.459 |
| **Total bilirubin, μmol/L** | 12.12 (10.37) | 11.41 (11.47) | 0.031 |  | 11.62 (9.97) | 11.41 (11.47) | 0.574 |

**Abbreviation:**

BMI, Body mass index; Neut, Neutrophil; Lymph, Lymphocyte; Glu, Glucose; HDL, High-density lipoprotein; LDL, Low-density lipoprotein; ALT, Alanine aminotransferase; AST, Aspartate aminotransferase; CREA, Creatinine; GFR, glomerular filtration rate; CRP, C–reactive protein; PCT, Procalcitonin; PT, Prothrombin time; APTT, Activated partial thromboplastin time; CH, Cholesterol; TG, Triglyceride; ALP, Alkaline phosphatase; GGT, Gamma-glutamyl transpeptidase; ALB, Albumin; TBIL, Total bilirubin.

**Table S2. Baseline characteristics of patients with SARS-CoV-2 infection before and after propensity score matching using Probit model from Henan cohort.**

| **Characteristics** | **Before matching** | | |  | **After 2:1 matching** | | |
| --- | --- | --- | --- | --- | --- | --- | --- |
|  | **Azvudine (n=6943)** | **Paxlovid (n=1202)** | **P value** |  | **Azvudine (n=6943)** | **Paxlovid (n=1202)** | **P value** |
| **Age, mean (SD), year** | 68.37 (15.22) | 69.26 (14.81) | 0.058 |  | 68.73 (14.36) | 69.26 (14.81) | 0.293 |
| **Gender, n (%)** |  |  | <0.001 |  |  |  | 0.97 |
| **Male** | 4173 (60.1) | 790 (65.7) |  |  | 1577 (65.6) | 790 (65.7) |  |
| **Female** | 2770 (39.9) | 412 (34.3) |  |  | 827 (34.4) | 412 (34.3) |  |
| **BMI, mean (SD), kg/m^2^** | 24.23 (3.94) | 24.44 (3.84) | 0.621 |  | 24.44 (4.07) | 24.44 (3.84) | 0.444 |
| **Severity at admission, n (%)** |  |  | <0.001 |  |  |  | 0.725 |
| **Mild** | 977 (14.1) | 57 (4.7) |  |  | 126 (5.2) | 57 (4.7) |  |
| **Moderate** | 4459 (64.2) | 740 (61.6) |  |  | 1491 (62.0) | 740 (61.6) |  |
| **Severe** | 1507 (21.7) | 405 (33.7) |  |  | 787 (32.7) | 405 (33.7) |  |
| **Vaccination doses, n (%)** |  |  | 0.088 |  |  |  | 0.906 |
| **0 dose** | 2228 (32.1) | 343 (28.5) |  |  | 670 (27.9) | 343 (28.5) |  |
| **1 dose** | 417 (6.0) | 74 (6.2) |  |  | 155 (6.4) | 74 (6.2) |  |
| **2 doses** | 921 (13.3) | 190 (15.8) |  |  | 365 (15.2) | 190 (15.8) |  |
| **3 doses** | 3313 (47.7) | 583 (48.5) |  |  | 1188 (49.4) | 583 (48.5) |  |
| **4 doses** | 62 (0.9) | 12 (1.0) |  |  | 26 (1.1) | 12 (1.0) |  |
| **5 doses** | 2 (0.0) | 0 (0.0) |  |  | 0 (0.0) | 0 (0.0) |  |
| **Time from diagnosis to treatment exposure, n (%)** |  |  | <0.001 |  |  |  | 0.11 |
| **> 5 days** | 1136 (16.4) | 409 (34.0) |  |  | 753 (31.3) | 409 (34.0) |  |
| **0–5 days** | 5807 (83.6) | 793 (66.0) |  |  | 1651 (68.7) | 793 (66.0) |  |
| **Concomitant antibiotics, n (%)** |  |  | <0.001 |  |  |  | 0.317 |
| **No** | 3070 (44.2) | 743 (61.8) |  |  | 1443 (60.0) | 743 (61.8) |  |
| **Yes** | 3873 (55.8) | 459 (38.2) |  |  | 961 (40.0) | 459 (38.2) |  |
| **Concomitant systemic steroid, n (%)** |  |  | <0.001 |  |  |  | 0.594 |
| **No** | 3875 (55.8) | 795 (66.1) |  |  | 1567 (65.2) | 795 (66.1) |  |
| **Yes** | 3068 (44.2) | 407 (33.9) |  |  | 837 (34.8) | 407 (33.9) |  |
| **Comorbidities, n (%)** |  |  |  |  |  |  |  |
| **Diabetes** | 1764 (25.4) | 322 (26.8) | 0.328 |  | 622 (25.9) | 322 (26.8) | 0.583 |
| **Hypertension** | 3106 (44.7) | 503 (41.8) | 0.067 |  | 1017 (42.3) | 503 (41.8) | 0.821 |
| **Liver diseases** | 770 (11.1) | 242 (20.1) | <0.001 |  | 460 (19.1) | 242 (20.1) | 0.503 |
| **Cardio-cerebral diseases** | 2196 (31.6) | 434 (36.1) | 0.002 |  | 813 (33.8) | 434 (36.1) | 0.185 |
| **Kidney diseases** | 1864 (26.8) | 247 (20.5) | <0.001 |  | 536 (22.3) | 247 (20.5) | 0.247 |
| **Primary malignant tumor** | 730 (10.5) | 147 (12.2) | 0.085 |  | 281 (11.7) | 147 (12.2) | 0.675 |
| **Chronic respiratory diseases** | 1164 (16.8) | 180 (15.0) | 0.133 |  | 365 (15.2) | 180 (15.0) | 0.908 |
| **Autoimmune diseases** | 234 (3.4) | 59 (4.9) | 0.0.1 |  | 112 (4.7) | 59 (4.9) | 0.803 |
| **Laboratory parameters, mean (SD)** |  |  |  |  |  |  |  |
| **Neutrophil, ×10^9^/L** | 5.81 (4.80) | 6.45 (4.74) | <0.001 |  | 6.21 (4.50) | 6.45 (4.74) | 0.129 |
| **Lymphocyte, ×10^9^/L** | 1.14 (2.37) | 1.17 (3.52) | 0.737 |  | 1.08 (1.61) | 1.17 (3.52) | 0.294 |
| **Glucose, mmol/L** | 8.02 (4.13) | 8.23 (4.26) | 0.103 |  | 8.23 (4.32) | 8.23 (4.26) | 0.986 |
| **High-density lipoprotein, mmol/L** | 1.16 (2.09) | 1.09 (1.30) | 0.269 |  | 1.09 (1.45) | 1.09 (1.30) | 0.987 |
| **Low-density lipoprotein, mmol/L** | 2.36 (2.26) | 2.28 (1.38) | 0.223 |  | 2.29 (2.04) | 2.28 (1.38) | 0.918 |
| **Alanine aminotransferase, IU/L** | 37.51 (101.01) | 40.71 (74.34) | 0.295 |  | 41.21 (128.04) | 40.71 (74.34) | 0.899 |
| **Aspartate aminotransferase, IU/L** | 42.83 (153.92) | 39.69 (52.09) | 0.485 |  | 41.46 (105.89) | 39.69 (52.09) | 0.584 |
| **Creatine, μmol/L** | 108.00 (180.11) | 97.70 (132.22) | 0.058 |  | 96.55 (127.71) | 97.70 (132.22) | 0.802 |
| **Glomerular filtration rate, ml/min** | 80.47 (55.31) | 84.34 (60.65) | 0.027 |  | 84.61 (73.34) | 84.34 (60.65) | 0.913 |
| **C-reactive protein, mg/L** | 53.98 (65.79) | 57.48 (64.84) | 0.088 |  | 58.74 (69.95) | 57.48 (64.84) | 0.602 |
| **Procalcitonin, ng/ml** | 1.24 (7.22) | 1.25 (6.67) | 0.954 |  | 1.49 (8.85) | 1.25 (6.67) | 0.408 |
| **Prothrombin time, s** | 16.95 (10.29) | 15.48 (10.47) | <0.001 |  | 15.63 (8.32) | 15.48 (10.47) | 0.65 |
| **Activated partial thromboplastin time, s** | 27.54 (12.18) | 28.22 (15.28) | 0.084 |  | 28.20 (11.69) | 28.22 (15.28) | 0.963 |
| **Cholesterol, mmol/L** | 4.01 (2.28) | 3.85 (1.13) | 0.022 |  | 3.89 (2.05) | 3.85 (1.13) | 0.587 |
| **Triglyceride, mmol/L** | 1.50 (2.42) | 1.41 (1.43) | 0.181 |  | 1.44 (2.05) | 1.41 (1.43) | 0.572 |
| **Alkaline phosphatase, IU/L** | 82.46 (52.57) | 87.71 (92.21) | 0.005 |  | 85.09 (59.55) | 87.71 (92.21) | 0.304 |
| **Gamma-glutamyl transpeptidase, IU/L** | 58.03 (125.51) | 59.65 (71.84) | 0.664 |  | 58.63 (80.48) | 59.65 (71.84) | 0.711 |
| **Albumin, g/L** | 36.76 (34.00) | 33.57 (8.77) | 0.001 |  | 32.72 (12.61) | 33.57 (8.77) | 0.036 |
| **Total bilirubin, μmol/L** | 12.19 (11.29) | 11.44 (12.31) | 0.037 |  | 11.60 (8.23) | 11.44 (12.31) | 0.637 |

**Abbreviation:**

BMI, Body mass index; Neut, Neutrophil; Lymph, Lymphocyte; Glu, Glucose; HDL, High-density lipoprotein; LDL, Low-density lipoprotein; ALT, Alanine aminotransferase; AST, Aspartate aminotransferase; CREA, Creatinine; GFR, glomerular filtration rate; CRP, C–reactive protein; PCT, Procalcitonin; PT, Prothrombin time; APTT, Activated partial thromboplastin time; CH, Cholesterol; TG, Triglyceride; ALP, Alkaline phosphatase; GGT, Gamma-glutamyl transpeptidase; ALB, Albumin; TBIL, Total bilirubin.

**Table S3. Baseline characteristics before and after propensity score matching in the Henan cohort of SARS-CoV-2 infection patients discharged within 1 day of receiving antiviral therapy were excluded.**

| **Characteristics** | **Before matching** | | |  | **After 2:1 matching** | | |
| --- | --- | --- | --- | --- | --- | --- | --- |
|  | **Azvudine (n=6824)** | **Paxlovid (n=1168)** | **P value** |  | **Azvudine (n=2336)** | **Paxlovid (n=1168)** | **P value** |
| **Age, mean (SD), year** | 68.30 (15.26) | 69.29 (14.79) | 0.04 |  | 69.08 (14.40) | 69.29 (14.79) | 0.685 |
| **Gender, n (%)** |  |  | <0.001 |  |  |  | 0.431 |
| **Male** | 4096 (60.0) | 767 (65.7) |  |  | 1501 (64.3) | 767 (65.7) |  |
| **Female** | 2728 (40.0) | 401 (34.3) |  |  | 835 (35.7) | 401 (34.3) |  |
| **BMI, mean (SD), kg/m^2^** | 24.18 (3.94) | 24.45 (3.86) | 0.032 |  | 24.41 (4.05) | 24.45 (3.86) | 0.77 |
| **Severity at admission, n (%)** |  |  | <0.001 |  |  |  | 0.457 |
| **Mild** | 971 (14.2) | 56 (4.8) |  |  | 131 (5.6) | 56 (4.8) |  |
| **Moderate** | 4417 (64.7) | 723 (61.9) |  |  | 1462 (62.6) | 723 (61.9) |  |
| **Severe** | 1436 (21.0) | 389 (33.3) |  |  | 743 (31.8) | 389 (33.3) |  |
| **Vaccination doses, n (%)** |  |  | 0.145 |  |  |  | 0.979 |
| **0 dose** | 2192 (32.1) | 335 (28.7) |  |  | 695 (29.8) | 335 (28.7) |  |
| **1 dose** | 416 (6.1) | 74 (6.3) |  |  | 144 (6.2) | 74 (6.3) |  |
| **2 doses** | 909 (13.3) | 182 (15.6) |  |  | 357 (15.3) | 182 (15.6) |  |
| **3 doses** | 3248 (47.6) | 565 (48.4) |  |  | 1116 (47.8) | 565 (48.4) |  |
| **4 doses** | 58 (0.8) | 12 (1.0) |  |  | 24 (1.0) | 12 (1.0) |  |
| **5 doses** | 1 (0.0) | 0 (0.0) |  |  | 0 (0.0) | 0 (0.0) |  |
| **Time from diagnosis to treatment exposure, n (%)** |  |  | <0.001 |  |  |  | 0.166 |
| **> 5 days** | 1133 (16.6) | 402 (34.4) |  |  | 748 (32.0) | 402 (34.4) |  |
| **0–5 days** | 5691 (83.4) | 766 (65.6) |  |  | 1588 (68.0) | 766 (65.6) |  |
| **Concomitant antibiotics, n (%)** |  |  | <0.001 |  |  |  | 0.932 |
| **No** | 3028 (44.4) | 720 (61.6) |  |  | 1435 (61.4) | 720 (61.6) |  |
| **Yes** | 3796 (55.6) | 448 (38.4) |  |  | 901 (38.6) | 448 (38.4) |  |
| **Concomitant systemic steroid, n (%)** |  |  | <0.001 |  |  |  | 0.99 |
| **No** | 3835 (56.2) | 779 (66.7) |  |  | 1556 (66.6) | 779 (66.7) |  |
| **Yes** | 2989 (43.8) | 389 (33.3) |  |  | 780 (33.4) | 389 (33.3) |  |
| **Comorbidities, n (%)** |  |  |  |  |  |  |  |
| **Diabetes** | 1725 (25.3) | 316 (27.1) | 0.211 |  | 624 (26.7) | 316 (27.1) | 0.861 |
| **Hypertension** | 3054 (44.8) | 490 (42.0) | 0.08 |  | 982 (42.0) | 490 (42.0) | 0.99 |
| **Liver diseases** | 761 (11.2) | 239 (20.5) | <0.001 |  | 446 (19.1) | 239 (20.5) | 0.358 |
| **Cardio-cerebral diseases** | 2151 (31.5) | 422 (36.1) | 0.002 |  | 818 (35.0) | 422 (36.1) | 0.541 |
| **Kidney diseases** | 1836 (26.9) | 241 (20.6) | <0.001 |  | 488 (20.9) | 241 (20.6) | 0.895 |
| **Primary malignant tumor** | 719 (10.5) | 145 (12.4) | 0.063 |  | 290 (12.4) | 145 (12.4) | 1 |
| **Chronic respiratory diseases** | 1140 (16.7) | 173 (14.8) | 0.116 |  | 346 (14.8) | 173 (14.8) | 1 |
| **Autoimmune diseases** | 231 (3.4) | 58 (5.0) | 0.01 |  | 113 (4.8) | 58 (5.0) | 0.934 |
| **Laboratory parameters, mean (SD)** |  |  |  |  |  |  |  |
| **Neutrophil, ×10^9^/L** | 5.77 (4.71) | 6.41 (4.70) | <0.001 |  | 6.29 (5.85) | 6.41 (4.70) | 0.555 |
| **Lymphocyte, ×10^9^/L** | 1.15 (2.44) | 1.22 (3.85) | 0.425 |  | 1.08 (1.63) | 1.22 (3.85) | 0.123 |
| **Glucose, mmol/L** | 8.00 (4.14) | 8.31 (4.22) | 0.017 |  | 8.23 (4.41) | 8.31 (4.22) | 0.606 |
| **High-density lipoprotein, mmol/L** | 1.17 (2.14) | 1.10 (1.61) | 0.309 |  | 1.08 (1.50) | 1.10 (1.61) | 0.749 |
| **Low-density lipoprotein, mmol/L** | 2.35 (2.15) | 2.29 (1.73) | 0.442 |  | 2.30 (1.82) | 2.29 (1.73) | 0.887 |
| **Alanine aminotransferase, IU/L** | 36.08 (93.32) | 39.72 (66.57) | 0.201 |  | 39.77 (71.70) | 39.72 (66.57) | 0.984 |
| **Aspartate aminotransferase, IU/L** | 42.44 (153.59) | 39.59 (52.37) | 0.53 |  | 40.11 (58.92) | 39.59 (52.37) | 0.8 |
| **Creatine, μmol/L** | 108.18 (179.85) | 96.69 (132.73) | 0.037 |  | 96.75 (136.83) | 96.69 (132.73) | 0.99 |
| **Glomerular filtration rate, ml/min** | 82.20 (60.41) | 82.49 (52.13) | 0.876 |  | 82.24 (57.55) | 82.49 (52.13) | 0.899 |
| **C-reactive protein, mg/L** | 54.24 (65.73) | 57.73 (64.87) | 0.093 |  | 57.84 (71.02) | 57.73 (64.87) | 0.963 |
| **Procalcitonin, ng/ml** | 1.25 (7.33) | 1.03 (5.21) | 0.339 |  | 1.19 (6.80) | 1.03 (5.21) | 0.48 |
| **Prothrombin time, s** | 16.81 (10.39) | 15.31 (9.22) | <0.001 |  | 15.28 (8.01) | 15.31 (9.22) | 0.918 |
| **Activated partial thromboplastin time, s** | 27.44 (11.13) | 27.73 (12.93) | 0.421 |  | 28.06 (10.47) | 27.73 (12.93) | 0.412 |
| **Cholesterol, mmol/L** | 4.05 (2.36) | 3.94 (1.93) | 0.148 |  | 3.93 (1.83) | 3.94 (1.93) | 0.868 |
| **Triglyceride, mmol/L** | 1.52 (2.59) | 1.42 (1.69) | 0.186 |  | 1.41 (1.94) | 1.42 (1.69) | 0.955 |
| **Alkaline phosphatase, IU/L** | 81.70 (50.53) | 87.48 (93.01) | 0.002 |  | 86.27 (60.23) | 87.48 (93.01) | 0.644 |
| **Gamma-glutamyl transpeptidase, IU/L** | 56.66 (105.72) | 60.92 (93.22) | 0.196 |  | 62.28 (145.11) | 60.92 (93.22) | 0.77 |
| **Albumin, g/L** | 36.59 (25.80) | 33.44 (8.89) | <0.001 |  | 33.20 (13.02) | 33.44 (8.89) | 0.569 |
| **Total bilirubin, μmol/L** | 11.98 (10.26) | 11.50 (12.29) | 0.152 |  | 11.53 (7.67) | 11.50 (12.29) | 0.936 |

**Abbreviation:**

BMI, Body mass index; Neut, Neutrophil; Lymph, Lymphocyte; Glu, Glucose; HDL, High-density lipoprotein; LDL, Low-density lipoprotein; ALT, Alanine aminotransferase; AST, Aspartate aminotransferase; CREA, Creatinine; GFR, glomerular filtration rate; CRP, C–reactive protein; PCT, Procalcitonin; PT, Prothrombin time; APTT, Activated partial thromboplastin time; CH, Cholesterol; TG, Triglyceride; ALP, Alkaline phosphatase; GGT, Gamma-glutamyl transpeptidase; ALB, Albumin; TBIL, Total bilirubin.

**Table S4. Baseline characteristics before and after propensity score matching in the Xinjiang cohort of SARS-CoV-2 infection patients.**

| **Characteristics** | **Before matching** | | |  | **After 2:1 matching** | | |
| --- | --- | --- | --- | --- | --- | --- | --- |
|  | **Azvudine (n=79)** | **Paxlovid (n=94)** | **P value** |  | **Azvudine (n=79)** | **Paxlovid (n=78)** | **P value** |
| **Age, mean (SD), year** | 69.03 (15.14) | 72.11 (16.71) | 0.209 |  | 69.03 (15.14) | 73.43 (16.32) | 0.081 |
| **Gender, n (%)** |  |  | 0.31 |  |  |  | 0.131 |
| **Male** | 47 (59.5) | 64 (68.1) |  |  | 47 (59.5) | 57 (72.2) |  |
| **Female** | 32 (40.5) | 30 (31.9) |  |  | 32 (40.5) | 22 (27.8) |  |
| **BMI, mean (SD), kg/m^2^** | 23.44 (3.88) | 23.91 (3.29) | 0.385 |  | 23.44 (3.88) | 23.86 (3.30) | 0.469 |
| **Severity at admission, n (%)** |  |  | 0.754 |  |  |  | 0.776 |
| **Mild** | 2 (2.5) | 1 (1.1) |  |  | 2 (2.5) | 1 (1.3) |  |
| **Moderate** | 43 (54.4) | 53 (56.4) |  |  | 43 (54.4) | 41 (51.9) |  |
| **Severe** | 34 (43.0) | 40 (42.6) |  |  | 34 (43.0) | 37 (46.8) |  |
| **Vaccination doses, n (%)** |  |  | 0.546 |  |  |  | 0.376 |
| **0 dose** | 37 (46.8) | 48 (51.1) |  |  | 37 (46.8) | 41 (51.9) |  |
| **1 dose** | 8 (10.1) | 8 (8.5) |  |  | 8 (10.1) | 6 (7.6) |  |
| **2 doses** | 5 (6.3) | 2 (2.1) |  |  | 5 (6.3) | 1 (1.3) |  |
| **3 doses** | 16 (20.3) | 24 (25.5) |  |  | 16 (20.3) | 21 (26.6) |  |
| **Unknown^a^** | 13 (16.5) | 12 (12.8) |  |  | 13 (16.5) | 10 (12.7) |  |
| **Time from diagnosis to treatment exposure, n (%)** |  |  | 0.366 |  |  |  | 0.16 |
| **> 5 days** | 14 (17.7) | 11 (11.7) |  |  | 14 (17.7) | 7 (8.9) |  |
| **0–5 days** | 65 (82.3) | 83 (88.3) |  |  | 65 (82.3) | 72 (91.1) |  |
| **Concomitant antibiotics, n (%)** |  |  | 0.177 |  |  |  | 0.108 |
| **No** | 72 (91.1) | 78 (83.0) |  |  | 72 (91.1) | 64 (81.0) |  |
| **Yes** | 7 (8.9) | 16 (17.0) |  |  | 7 (8.9) | 15 (19.0) |  |
| **Concomitant systemic steroid, n (%)** |  |  | 0.455 |  |  |  | 0.302 |
| **No** | 58 (73.4) | 63 (67.0) |  |  | 58 (73.4) | 51 (64.6) |  |
| **Yes** | 21 (26.6) | 31 (33.0) |  |  | 21 (26.6) | 28 (35.4) |  |
| **Comorbidities, n (%)** |  |  |  |  |  |  |  |
| **Diabetes** | 27 (34.2) | 37 (39.4) | 0.585 |  | 27 (34.2) | 34 (43.0) | 0.327 |
| **Hypertension** | 44 (55.7) | 45 (47.9) | 0.383 |  | 44 (55.7) | 36 (45.6) | 0.265 |
| **Liver diseases** | 32 (40.5) | 25 (26.6) | 0.076 |  | 32 (40.5) | 20 (25.3) | 0.063 |
| **Cardio-cerebral diseases** | 50 (63.3) | 60 (63.8) | 1 |  | 50 (63.3) | 49 (62.0) | 1 |
| **Kidney diseases** | 18 (22.8) | 31 (33.0) | 0.189 |  | 18 (22.8) | 29 (36.7) | 0.082 |
| **Primary malignant tumor** | 14 (17.7) | 25 (26.6) | 0.227 |  | 14 (17.7) | 22 (27.8) | 0.184 |
| **Chronic respiratory diseases** | 19 (24.1) | 16 (17.0) | 0.339 |  | 19 (24.1) | 11 (13.9) | 0.156 |
| **Autoimmune diseases** | 6 (7.6) | 4 (4.3) | 0.541 |  | 6 (7.6) | 2 (2.5) | 0.276 |
| **Laboratory parameters, mean (SD)** |  |  |  |  |  |  |  |
| **Neutrophil, ×10^9^/L** | 5.73 (4.26) | 6.20 (4.31) | 0.476 |  | 5.73 (4.26) | 6.48 (4.44) | 0.285 |
| **Lymphocyte, ×10^9^/L** | 0.90 (0.60) | 0.94 (1.50) | 0.81 |  | 0.90 (0.60) | 0.93 (1.63) | 0.892 |
| **Glucose, mmol/L** | 8.43 (4.83) | 8.51 (5.01) | 0.915 |  | 8.43 (4.83) | 8.58 (4.82) | 0.849 |
| **High-density lipoprotein, mmol/L** | 0.82 (0.25) | 0.82 (0.26) | 0.939 |  | 0.82 (0.25) | 0.82 (0.27) | 0.865 |
| **Low-density lipoprotein, mmol/L** | 2.25 (0.93) | 2.14 (0.79) | 0.396 |  | 2.25 (0.93) | 2.10 (0.78) | 0.273 |
| **Alanine aminotransferase, IU/L** | 116.21 (409.86) | 54.97 (52.18) | 0.153 |  | 116.21 (409.86) | 56.22 (51.43) | 0.199 |
| **Aspartate aminotransferase, IU/L** | 43.14 (107.58) | 39.55 (38.09) | 0.763 |  | 43.14 (107.58) | 41.79 (38.58) | 0.916 |
| **Creatine, μmol/L** | 119.53 (179.79) | 114.52 (114.57) | 0.825 |  | 119.53 (179.79) | 118.17 (115.05) | 0.955 |
| **Glomerular filtration rate, ml/min** | 70.59 (24.98) | 74.08 (26.48) | 0.376 |  | 70.59 (24.98) | 75.87 (25.39) | 0.189 |
| **C-reactive protein, mg/L** | 39.97 (33.81) | 47.01 (33.29) | 0.171 |  | 39.97 (33.81) | 51.39 (33.02) | 0.033 |
| **Procalcitonin, ng/ml** | 0.77 (1.92) | 0.93 (2.99) | 0.683 |  | 0.77 (1.92) | 1.08 (3.24) | 0.472 |
| **Prothrombin time, s** | 20.71 (2.79) | 20.14 (2.85) | 0.188 |  | 20.71 (2.79) | 20.19 (2.75) | 0.235 |
| **Activated partial thromboplastin time, s** | 31.86 (6.28) | 30.82 (4.45) | 0.206 |  | 31.86 (6.28) | 30.53 (4.18) | 0.121 |
| **Cholesterol, mmol/L** | 3.66 (0.92) | 3.41 (0.92) | 0.076 |  | 3.66 (0.92) | 3.39 (0.89) | 0.065 |
| **Triglyceride, mmol/L** | 1.24 (0.43) | 1.33 (0.54) | 0.218 |  | 1.24 (0.43) | 1.34 (0.54) | 0.197 |
| **Alkaline phosphatase, IU/L** | 123.01 (158.25) | 85.93 (48.71) | 0.033 |  | 123.01 (158.25) | 85.11 (39.86) | 0.041 |
| **Gamma-glutamyl transpeptidase, IU/L** | 93.27 (159.84) | 65.29 (81.36) | 0.14 |  | 93.27 (159.84) | 65.30 (68.16) | 0.155 |
| **Albumin, g/L** | 32.29 (5.09) | 32.93 (5.10) | 0.407 |  | 32.29 (5.09) | 32.68 (4.36) | 0.601 |
| **Total bilirubin, μmol/L** | 19.75 (24.80) | 16.02 (11.80) | 0.197 |  | 19.75 (24.80) | 15.40 (5.19) | 0.129 |

**Abbreviation:**

BMI, Body mass index; Neut, Neutrophil; Lymph, Lymphocyte; Glu, Glucose; HDL, High-density lipoprotein; LDL, Low-density lipoprotein; ALT, Alanine aminotransferase; AST, Aspartate aminotransferase; CREA, Creatinine; GFR, glomerular filtration rate; CRP, C–reactive protein; PCT, Procalcitonin; PT, Prothrombin time; APTT, Activated partial thromboplastin time; CH, Cholesterol; TG, Triglyceride; ALP, Alkaline phosphatase; GGT, Gamma-glutamyl transpeptidase; ALB, Albumin; TBIL, Total bilirubin.

**^a^**: number of vaccinations≥ 1 time.

**Table S5. Incidence of adverse event of COVID-19 patients receiving azvudine and Paxlovid in Henan cohort.**

| **Adverse events (n, %)** | **Available data^a^** | |  | **Grade 1^b^** | | |  | **Grade 2** | | |  | **Grade ≥ 3** | | |
| --- | --- | --- | --- | --- | --- | --- | --- | --- | --- | --- | --- | --- | --- | --- |
|  | **Azvudine** | **Paxlovid** |  | **Azvudine** | **Paxlovid** | **P value** |  | **Azvudine** | **Paxlovid** | **P value** |  | **Azvudine** | **Paxlovid** | **P value** |
| **Decreased lymphocyte count** | 2093 | 95 |  | 97 (4.6%) | 35 (3.7%) | **0.2** |  | 203 (9.7%) | 90 (9.4%) | **0.8** |  | 418 (20%) | 249 (26%) | **<0.001** |
| **Increased lymphocyte count** | 2093 | 95 |  | 24 (1.1%) | 12 (1.3%) | **0.8** |  | 21 (1.0%) | 4 (0.4%) | **0.1** |  | 4 (0.2%) | 2 (0.2%) | **>0.9** |
| **Increased neutrophil count** | 1104 | 714 |  | 16 (1.4%) | 13 (1.8%) | **0.5** |  | 22 (2.0%) | 9 (1.3%) | **0.2** |  | 14 (1.3%) | 13 (1.8%) | **0.3** |
| **Decreased PLT count** | 1455 | 794 |  | 61 (4.2%) | 43 (5.4%) | **0.2** |  | 31 (2.1%) | 32 (4.0%) | **0.009** |  | 72 (4.9%) | 53 (6.7%) | **0.088** |
| **Anemia** | 1139 | 731 |  | 200 (18%) | 115 (16%) | **0.3** |  | 142 (12%) | 90 (12%) | **>0.9** |  | 110 (9.7%) | 82 (11%) | **0.3** |
| **Hypophosphatemia^c^** | 668 | 552 |  | 107 (16%) | 116 (21%) | **0.025** |  |  |  |  |  |  |  |  |
| **Hypokalemia^d^** | 1688 | 903 |  |  |  |  |  | 245 (15%) | 115 (13%) | **0.2** |  | 147 (8.7%) | 69 (7.6%) | **0.3** |
| **Hyperkalemia^d^** | 1835 | 986 |  |  |  |  |  | 58 (3.4%) | 31 (3.4%) | **>0.9** |  | 13 (0.8%) | 2 (0.2%) | **0.079** |
| **Increased ALT** | 1315 | 726 |  | 269 (20%) | 183 (25%) | **0.013** |  | 31 (2.4%) | 29 (4.0%) | **0.036** |  | 30 (2.3%) | 22 (3.0%) | **0.3** |
| **Increased AST** | 1377 | 745 |  | 209 (15%) | 138 (19%) | **0.047** |  | 26 (1.9%) | 17 (2.3%) | **0.5** |  | 32 (2.3%) | 22 (3.0%) | **0.4** |
| **Increased ALP** | 1295 | 742 |  | 87 (6.7%) | 49 (6.6%) | **>0.9** |  | 10 (0.8%) | 6 (0.8%) | **>0.9** |  | 0 (0%) | 1 (0.1%) | **0.4** |
| **Increased GGT** | 966 | 684 |  | 122 (13%) | 104 (15%) | **0.13** |  | 30 (3.1%) | 26 (3.8%) | **0.4** |  | 8 (0.8%) | 8 (1.2%) | **0.5** |
| **Hyperuricemia^c^** | 1117 | 683 |  | 74 (6.6%) | 39 (5.7%) | **0.4** |  |  |  |  |  |  |  |  |
| **Increased CREA** | 1389 | 740 |  | 49 (3.5%) | 33 (4.5%) | **0.3** |  | 37 (2.7%) | 34 (4.6%) | **0.018** |  | 33 (2.4%) | 15 (2.0%) | **0.6** |
| **Hypoglycemia^c^** | 529 | 77 |  | 93 (18%) | 14 (18%) | **0.9** |  |  |  |  |  |  |  |  |
| **Hypercholesterolemia** | 318 | 126 |  | 17 (5.3%) | 23 (18%) | **<0.001** |  | 2 (0.6%) | 0 (0%) | **>0.9** |  | 0 (0%) | 1 (0.8%) | **0.3** |
| **Hypertriglyceridemia** | 240 | 111 |  | 39 (16%) | 20 (18%) | **0.7** |  | 5 (2.1%) | 4 (3.6%) | **0.5** |  | 2 (0.8%) | 1 (0.9%) | **>0.9** |

**Abbreviations:** PLT, platelets; Hb, hemoglobin; ALT, alanine aminotransferase; AST, aspartate aminotransferase; ALP, alkaline phosphatase; UA, uric acid; CREA, creatinine; Glu, glucose.

**^a^:** Number of people who completed the follow-up of data collection for this indicator.

**^b^**: Severity grades were defined according to the National Cancer Institute Common Terminology Criteria for Adverse Events (CTCAE), version 5.0.

**^c^**: There is no grade for this adverse event, only with or without it.

**^d^:** The adverse event was classified as Grade ≤ 2 and ≥ 3.

**Study Protocol for "** **Real-world effectiveness and safety of oral azvudine versus nirmatrelvir‒ritonavir (Paxlovid) in hospitalized patients with COVID-19: A multicenter, retrospective, cohort study"**

| Version | V1.0 |
| --- | --- |
| Edition date | 2023.08.27 |
| Principal investigator | Zujiang Yu |
| Study performing organization | The First Affiliated Hospital of Zhengzhou University |
| Participating organization | Henan Provincial Chest Hospital  Henan Provincial Infectious Disease Hospital  Anyang City Fifth People's Hospital  Shangqiu Municipal Hospital  Nanyang Central Hospital  Luoyang Central Hospital Affiliated of Zhengzhou University  Guangshan County People's Hospital  Fengqiu County People's Hospital  the First Affiliated Hospital of Henan University of Science & Technology  the First Affiliated Hospital of Xinjiang Medical University |
| Sponsor | The First Affiliated Hospital of Zhengzhou University |

**Confidentiality Statement**

This study protocol is confidential information intended to be provided to medical experts related to this study, researchers participating in this study and other study-related staff, as well as relevant business entrusting agencies such as medical institutions, ethics committees, and contract study organizations undertaking this study. Except for explaining the situation to the subjects, no content of this study protocol shall be disclosed or leaked to third parties without the prior written consent of the sponsor. In addition, when some or all of the results of this clinical study are published externally to societies, magazines, etc., written consent from the sponsor is required.

**Signature page**

**Real-world effectiveness and safety of oral azvudine versus nirmatrelvir‒ritonavir (Paxlovid) in hospitalized patients with COVID-19: A multicenter, retrospective, cohort study**

**Statement and Signature of Relevant Personnel**

1. Sponsor

We have participated in the discussion and revision of this clinical study protocol and agree to conduct clinical studys in accordance with it. During the clinical study process, we will conscientiously perform the sponsor's responsibilities by the current "Measures for the Administration of Drug Registration" and China's GCP and other relevant regulations.

Sponsor: The First Affiliated Hospital of Zhengzhou University

Project manager：

Signature of project manager:

Date：

**Signature page**

**Real-world effectiveness and safety of oral azvudine versus nirmatrelvir‒ritonavir (Paxlovid) in hospitalized patients with COVID-19: A multicenter, retrospective, cohort study**

**Statement and Signature of Relevant Personnel**

1. Study center

We have carefully discussed and revised the clinical study plan for this project, and will implement the clinical study in compliance with the provisions of this clinical study protocol. During the clinical study process, we will conscientiously perform the researcher's responsibilities by the current " Measures for the Administration of Drug Registration" and China's GCP and the Declaration of Helsinki.

**Study center：**

Principal investigator：

Signature of principal investigator：

Date：

Contents

[Abstract of protocol 5](#_Toc187507760)

[1 Study background 7](#_Toc187507761)

[2 Study objective 8](#_Toc187507762)

[3 Study design 8](#_Toc187507763)

[3.1 Overall design 8](#_Toc187507764)

[4 Subject selection 9](#_Toc187507765)

[4.1 Inclusion Criteria 9](#_Toc187507766)

[4.2 Exclusion criteria 9](#_Toc187507767)

[5 Study related drugs 9](#_Toc187507768)

[5.1 Research Drugs 9](#_Toc187507769)

[6 Research procedures 10](#_Toc187507770)

[6.1 Data Sources 10](#_Toc187507771)

[6.2 Definition of baseline variables 10](#_Toc187507772)

[6.3 Exposure Treatment 11](#_Toc187507773)

[6.4 Clinical Outcome 11](#_Toc187507774)

[6.5 Calculation of survival data 11](#_Toc187507775)

[6.6 Safety Evaluation 12](#_Toc187507776)

[7 Statistical Analysis 12](#_Toc187507777)

[7.1 Statistical Hypotheses and sample size 12](#_Toc187507778)

[7.2 General Principles 13](#_Toc187507779)

[7.3 Screening and matching of subjects 13](#_Toc187507780)

[7.4 Processing and analysis of missing data of baseline characteristics 14](#_Toc187507781)

[7.5 Analysis of efficacy 14](#_Toc187507782)

[7.6 Safety Analysis 15](#_Toc187507783)

[8 Research Management 15](#_Toc187507784)

[8.1 Ethics Committee 15](#_Toc187507785)

[8.2 Preservation of data 15](#_Toc187507786)

[8.3 Research Approval 15](#_Toc187507787)

# Abstract of protocol

| **Study title:** | **Real-world effectiveness and safety of oral azvudine versus nirmatrelvir‒ritonavir (Paxlovid) in hospitalized patients with COVID-19: A multicenter, retrospective, cohort study** |
| --- | --- |
| **Drug name** | **Azvudine** |
| **Study objective** | The effectiveness and safety of azvudine compared with Paxlovid in the treatment of COVID-19 |
| **Study design** | Retrospective study |
| **Subject** | COVID-19 patients |
| Sample size | Plans to enroll 40,000 subjects |
| **Subject selection** | **1. Inclusion criteria**：  Subjects must meet all the following standards to enter the study:   1. Aged 18 years or older, no gender limit; 2. Patients diagnosed with COVID-19, meeting diagnostic criteria in the latest version of COVID-19 diagnosis and treatment plan issued by the National Health Commission of China; 3. Patients hospitalized between December 5, 2022 and January 31, 2023.   **2. Exclusion criteria：**  Subjects who meet any of the following criteria will not be allowed to enter the study：   1. Patients who are known or suspected to be allergic to the components of azvudine or Paxlovid; 2. Patients with severe liver, kidney, heart and other organ damage; 3. Pregnant or lactating women; 4. Patients who are concurrently treated with both azvudine and Paxlovid or patients receiving other antiviral agents;   （5）Patients who did not receive any antiviral agents; |
| **Study drugs** | Name: Azvudine (FNC)  Specification: 1mg/tablet  Name: Nirmatrelvir/ritonavir (Paxlovid)  Specification: Nirmatrelvir tablets 150mg/tablet, ritonavir tablets 100mg/tablet  Storage conditions: sealed and stored at room temperature |
| **Study protocol** | A multicenter retrospective clinical study design was used to screen hospitalized patients with COVID-19 who met the criteria. The experimental group and the control group were matched 2:1 by propensity score. The experimental group received azvudine, and the control group received Nirmatrelvir/ritonavir. The all-cause death and composite disease progression of patients with COVID-19 were observed. Sensitivity analysis was used to further verify the study results. Safety evaluation was used to evaluate the effect of azvudine on the occurrence of adverse safety events. |
| **Efficacy indicator** | Primary outcome: all-cause death  Secondary outcome: composite disease progression |
| **Safety indicator** | Laboratory test results |

**Real-world Clinical Study of Azvudine for the Treatment of COVID-19**

# 1 Study background

The coronavirus disease 2019 (COVID-19) can cause respiratory symptoms, fever, cough, shortness of breath and dyspnea in humans. In critical cases, the virus mainly causes severe systemic inflammatory response (cytokine storm), causing acute lung injury, severe pneumonia, acute respiratory distress syndrome (ARDS), severe hypoxemia, septic shock, renal failure, and even death. The virus is highly contagious. The 2019-nCoV infection has spread rapidly around the world and has become a major threat to human health.

First-in-class new drug azvudine is a new nucleoside reverse transcriptase inhibitor. According to Roche, vitro tests have proven that azvudine can significantly inhibit RNA viruses, such as hepatitis C virus, Hand, foot and mouth virus, African swine fever virus and hepatitis B virus, and can significantly inhibit HIV, and have completed anti-HIV phase II clinical trials (GQ-AZVUDINE-201) and phase III trials, with good human efficacy and safety. The above data prove that azvudine has a significant and broad-spectrum inhibitory effect on RNA and DNA viruses, and has good clinical safety. This research team once published an article describing the effective results of azvudine in treating COVID-19 and published it in the journal Advanced Science.

In February 2022, Paxlovid received conditional emergency approval in China for the treatment of adult patients with mild to moderate COVID-19 who are at high risk of developing severe disease. Nirmatrelvir is a peptide inhibitor of the main protease of SARS-CoV-2 (namely the 3-chymotrypsin-like cysteine protease), which can prevent the enzyme from catalyzing the cleavage of viral polyproteins into non-structural proteins necessary for viral replication, thereby inhibiting viral replication. When used in combination with Nirmatrelvir, ritonavir can inhibit CYP3A-mediated metabolism of Nirmatrelvir, thereby increasing its blood concentration. A Phase II/III clinical trial (EPIC-HR) of non-hospitalized adult COVID-19 patients with a higher risk of severe progression showed that Paxlovid can reduce the risk of COVID-19-related hospitalization or death by 89% compared with placebo in patients treated within 3 days of symptom onset; in patients treated within 5 days of symptom onset, the risk of hospitalization or death was reduced by 88%. A retrospective cohort study showed that Paxlovid reduced the risk of death by 66%, the risk of hospitalization by 34%, and the risk of disease progression during hospitalization by 43% compared with patients who did not use antiviral drugs.

After the adjustment of epidemic prevention policies, a large-scale wave of COVID-19 occurred in China. During the first wave of the epidemic in December 2022, azvudine and Paxlovid were the key antiviral drugs recommended in the "Diagnosis and Treatment Plan for Pneumonia Infected by New Coronavirus (Trial Ninth or Tenth Edition)" published by the National Health Commission. Although a large number of studies have confirmed the effectiveness and safety of Paxlovid or azvudine, the results of comparative analysis of the effectiveness and safety of azvudine and Paxlovid remain controversial. Several studies have shown that treatment with azvudine is associated with a lower risk of composite disease progression and all-cause death compared with Paxlovid in hospitalized patients. In contrast, a multicenter retrospective cohort study showed no significant difference in all-cause death or composite disease progression between patients treated with azvudine and those treated with Paxlovid. Another study showed that treatment with azvudine was similarly effective as Paxlovid in reducing composite outcomes and short-term all-cause death in elderly patients with severe COVID-19. Therefore, a multi-center, retrospective cohort study is urgently needed to evaluate the effectiveness and safety of azvudine compared with Paxlovid in the treatment of hospitalized patients with COVID-19.

#

# 2 Study objective

The effectiveness and safety of azvudine compared with Paxlovid in the treatment of COVID-19.

# 3 Study design

## 3.1 Overall design

This study is a multicenter retrospective cohort study that collected hospitalized patients with COVID-19 from 10 hospitals in Henan Province from December 5, 2022 to January 31, 2023. Eligible patients were screened by inclusion and exclusion criteria and enrolled in the control group taking Paxlovid and the experimental group taking azvudine. The baseline data of the patients were used to match the experimental group and the control group using 1:2 propensity score matching. The efficacy of azvudine in the treatment of COVID-19 were evaluated by comparing the all-cause death and the incidence of composite disease progression.

**4 Subject selection**

The investigator must ensure that all participants in the study meet the following inclusion criteria and do not meet any of the exclusion criteria.

**4.1 Inclusion Criteria**

All subjects should meet the following criteria to enter the study:

(1) At least 18 years of age, regardless of gender;

(2) The patients are confirmed to be infected with the COVID-19 and meet the diagnostic criteria in the latest version of the "Diagnosis and Treatment Protocol for COVID-19" issued by the National Health Commission of China;

(3) Hospitalized patients from December 5, 2022 to January 31, 2023.

**4.2 Exclusion criteria**

Subjects who meet any of the following criteria will not be admitted to the study:

1. A known or suspected allergy to the components of azvudine tablets or Nirmatrelvir/ritonavir tablets;
2. Patients with severe liver, kidney, heart and other organ damage;
3. Pregnant or lactating women;
4. Patients treated with both azvudine tablets and Nirmatrelvir/ritonavir tablets at the same time or receiving other antiviral therapy;
5. Patients who did not receive any antiviral therapy;

**5 Study related drugs**

**5.1 Research Drugs**

Drug name, specification, packaging

Generic name: Azvudine tablets (Azvudine, FNC)

Dosage form: Tablet

Specification: 1mg/tablet

Storage conditions: Sealed and stored at room temperature.

Validity period: One year

Generic name: Nirmatrelvir/Ritonavir tablets (Paxlovid)

Dosage form: Tablet

Specification: Nirmatrelvir tablets 150mg/tablet, Ritonavir tablets 100mg/tablet

Storage conditions: Sealed and stored at room temperature

Validity period: One year

**6 Research procedures**

**6.1 Data Sources**

Electronic case records of all COVID-19 patients were retrieved from the inpatient systems of various hospitals. These records included demographic characteristics, admission data, diagnosis, prescription and medication records, laboratory tests, examinations, nursing notes, course of illness records, and discharge or death.

**6.2 Definition of baseline variables**

The baseline variables for this study include:

- Demographic and general baseline characteristics: age, sex, body mass index (BMI), severity of COVID-19, onset of admission accompanied by systemic steroids, onset of admission accompanied by antibiotic therapy, and number of vaccinations;
- Baseline comorbidity: diabetes, hypertension, liver disease, cardiovascular and cerebrovascular diseases, kidney disease, primary malignant tumor, chronic respiratory disease, and autoimmune disease;
- Baseline laboratory results: absolute neutrophil count, absolute lymphocyte count, glucose, high-density lipoprotein, low-density lipoprotein, glutamic pyruvic transaminase, glutamic oxaloacetic transaminase, creatinine, glomerular filtration rate, C-reactive protein, procalcitonin, plasma prothrombin time, activated partial thromboplastin time, total cholesterol, triglycerides, alkaline phosphatase, gamma-glutamyl transferase, albumin, and total bilirubin.

Baseline data were obtained from the patient's latest exam from the time of diagnosis to the time before medication.

The classification of COVID-19 is defined according to the "Diagnosis and Treatment Protocol for COVID-19 (Trial Version 10)" guidelines, and the severe and critical types in the guidelines were defined as severe in this study.

**6.3 Exposure Treatment**

Hospitalized patients with COVID-19 who received azvudine or Nirmatrelvir/ritonavir during the observation period were defined as exposure therapy.

**6.4 Clinical Outcome**

The primary efficacy endpoint was the time to all-cause death within 31 days after diagnosis of COVID-19.

The secondary efficacy endpoint was the time of composite disease progression within 31 days after diagnosis of COVID-19.

The safety endpoint was the results of laboratory tests.

Composite disease progression was defined as non-severe COVID-19 patients progress to severe or die, and death in all severity of COVID-19 patients. Composite disease progression may include the following: shortness of breath, RR≥30 beats/min; resting state, oxygen saturation ≤93% when inhaling air; arterial partial pressure of oxygen (PaO2)/oxygen absorption concentration (FiO2) ≤300mmHg (1mmHg=0.133kPa); the clinical symptoms got progressively worse, and the lung imaging showed that the lesion progressed significantly > 50% within 24 ~ 48 hours; high flow oxygen therapy; respiratory failure and mechanical ventilation (ventilator, tracheal intubation, extracorporeal membrane oxygenation (ECMO); Shock; admission to the intensive care unit (ICU); death.

**6.5 Calculation of survival data**

Survival analysis was used for time to all-cause death and the time of occurrence of composite disease progression, and time-to-event was calculated by the time elapsed from the start time point to the time point at which the event of concern occurred.

All-cause death time = Date of all-cause death — Date of COVID-19 diagnosis + 1d;

Time of composite disease progression = Date of composite disease progression — Date of diagnosis of COVID-19 + 1d.

The cut-off point for survival analysis of both efficacy endpoints was 31 days after COVID-19 diagnosis. If subjects did not experience all-cause death/composite disease progression events within 31 days, censoring was performed, and the censoring time was 31 days. If the subject was lost to follow-up, the time of last observation of the subject was used as the censoring time.

**6.6 Safety Evaluation**

The severity of the adverse events was judged by the NCI CTCAE 5.0 criteria. If unlisted adverse events occur, the severity of AE should also be described according to the guidelines proposed in NCI CTCAE 5.0. The guidelines are shown in Table 1:

**Table 1. Criteria for determining adverse events**

| **Adverse Event classification** | **Severity description** |
| --- | --- |
| I level | Mild, no clinical or mild clinical symptoms; only clinical or laboratory abnormalities; no treatment required. |
| II level | Moderate, requiring minimal, local or non-intrusive treatment; age-appropriate limited activities of daily living (Activities of Daily Living, ADL), daily use refers to cooking, shopping, phone calls, financial management, etc. |
| Ⅲ level | Serious or medically significant but not immediately life threatening; cause hospitalization or prolonged hospitalization; disability; restricted daily self-care (Self care ADL). Self-care in daily life refers to: bathing, dressing, stripping, eating, washing taking medicine, etc., non-bedridden. |
| IV level | Life-threatening and requires emergency treatment |
| V level | Death due to an adverse event. |

**7 Statistical Analysis**

**7.1 Statistical Hypotheses and sample size**

7.1.1 Statistical Hypotheses and test criteria

To test whether the efficacy of azvudine tablets is significantly better than conventional treatment, the Hazard Ratio (HR) of time to all-cause death within 31 days of confirmed COVID-19 (with control group as reference) was used to establish the following statistical hypotheses:

Null hypothesis H0: HR ≥ 1;

Alternative hypothesis H1: HR < 1.

The one-sided significance level was set at 0.025. The two-side p value was output during statistical analysis, and if p<0.05, H0 was rejected and H1 was accepted, that is, it was determined that the efficacy of azvudine tablets was significantly better than conventional treatment.

7.1.2 Calculation of sample size

This study was a retrospective study, and the sample size was not calculated by statistical hypothesis testing.

**7.2 General Principles**

The statistical analysis software is R® (4.4.1 or later). The results of the statistical analysis were reported in tables and graphics report.

7.2.1 Descriptive statistics

Descriptive statistics were performed on each variable for subjects in the experimental group and the control group.

Descriptive statistics of measurement data include mean and standard deviation;

The counting data will be summarized as the number of cases and percentage.

7.2.2 General Test

For endpoints other than validity endpoints (i.e. baseline variables and safety endpoints), appropriate statistical methods are used to test according to the variable types, and two-side P-value is provided:

Measurement data: T test was used when individual data satisfied normal distribution, and Wilcoxon rank sum test was used when individual data did not meet normal distribution.

Counting data: Chi-square test was used;

Rank data: Wilcoxon rank-sum test.

**7.3 Screening and matching of subjects**

The "MatchIt" package in R® was used to perform propensity score matching on patient data to obtain samples for statistical analysis. Propensity score matching was performed using the Logistic regression model combined with greedy matching method. Patient data were derived from the data of patients with COVID-19 who were hospitalized from 10 hospitals in Henan Province from December 5, 2022 to January 31, 2023. All patients who met the inclusion criteria and did not meet any exclusion criteria were screened, and then the samples were obtained for statistical analysis by propensity score matching. All baseline variables were selected as important covariates to participate in propensity score matching, and suitable subjects were included for the experimental group and the control group to maintain the balance of baseline characteristics.

**7.4 Processing and analysis of missing data of baseline characteristics**

Multiple Imputation (MI) method based on Missing at Random (MAR) is adopted for the missing data processing of baseline features.

Each baseline feature was analyzed and described based on the subjects before and after screening and matching. The description of the analysis was analyzed according to the descriptive statistical methods in the general principles.

7.4.1 Demographic and general baseline characteristics

Demographic data and general baseline characteristics were analyzed and summarized in groups of experimental and control groups. Measurement data and enumeration data were analyzed according to descriptive statistical methods in the general principles, respectively.

**7.5 Analysis of efficacy**

7.5.1 Analysis of primary and secondary efficacy endpoint

Firstly, survival analysis was performed by Kaplan-Meier method, survival functions were calculated and presented in the form of survival curves, and differences in survival functions or curves between groups were compared by Log-rank test. At the same time, a Cox proportional hazard regression model was established, taking the group as the independent variable and all baseline variables as the covariates to calculate the inter-group HR (control group as the reference) and its 95% CI.

7.5.3 Subgroup analysis

Patients were grouped according to the level of baseline characteristics, and the above analysis methods were repeated for primary and secondary efficacy endpoint analysis.

7.5.2 Sensitivity analysis

In order to explore the robustness of the main analysis method, the following three sensitivity analyses were performed using different analytical models: the first method was to use the average of existing data to replace the multiple random interpolation method in the main analysis to fill in missing data in baseline features. The second method was to perform propensity score matching using the Probit regression model combined with greedy matching method. The third was to exclude participants who were discharged from the hospital within one day of taking azvudine or Paxlovid orally.

**7.6 Safety Analysis**

Make descriptive statistics of abnormal changes in laboratory test results by enumeration data. Percentages were calculated using the number of analyzable cases in the treatment group as the denominator. The analysis included: decreased lymphocyte count; increased lymphocyte count; increased neutrophils count; decreased platelet count; decreased hemoglobin; abnormal blood phosphorus; hypokalemia; hyperkalemia; elevated alanine aminotransferase; increased glutamic oxal aminotransferase; increased alkaline phosphatase; increased glutamyl transpeptidase; increased blood uric acid; increased creatinine; hypoglycemia; high cholesterol; high triglycerides.

**8 Research Management**

**8.1 Ethics Committee**

Prior to commencement of the study, the investigator/research unit should obtain the Ethics Committee's approval of the study protocol, informed consent, and any other documents that may be required.

During the study period, any new amendments to the protocol, informed consent, etc., should be subject to ethical approval or filing.

**8.2 Preservation of data**

Study data should be retained until 5 years after the end of the study, but for a longer period if required by current legislation or agreement with the sponsor. The sponsor will notify the investigator in writing when the data will no longer be required to be kept.

**8.3 Research Approval**

The principal investigator and sponsor of the research unit should sign the signature page of this protocol, attesting that he/she has agreed to the content of this protocol and will conduct and implement the research in accordance with this protocol.
